# Incorporation of drug efflux inhibitor and chemotherapeutic agent into an inorganic/organic platform for the effective treatment of multidrug resistant breast cancer

**DOI:** 10.1186/s12951-019-0559-y

**Published:** 2019-12-23

**Authors:** Yang Dong, Hongze Liao, Jian Yu, Hao Fu, De Zhao, Ke Gong, Qi Wang, Yourong Duan

**Affiliations:** 1 State Key Laboratory of Oncogenes and Related Genes, Shanghai Cancer Institute, Renji Hospital, School of Medicine, Shanghai Jiao Tong University, Shanghai, 200032 China; 2 Marine Drugs Research Center, Department of Pharmacy, State Key Laboratory of Oncogenes and Related Genes, Renji Hospital, School of Medicine, Shanghai Jiao Tong University, Shanghai, 200127 China; 3 Key Laboratory for Advanced Materials and Institute of Fine Chemicals, Shanghai Key Laboratory of Functional Materials Chemistry, School of Chemistry and Molecular Engineering, East China University of Science and Technology, Shanghai, 200237 China

**Keywords:** Codelivery, MDR, Inorganic/organic, Verapamil, Novantrone

## Abstract

**Background:**

Multidrug resistance (MDR) is a pressing obstacle in clinical chemotherapy for breast cancer. Based on the fact that the drug efflux is an important factor in MDR, we designed a codelivery system to guide the drug efflux inhibitor verapamil (VRP) and the chemotherapeutic agent novantrone (NVT) synergistically into breast cancer cells to reverse MDR.

**Results:**

This co-delivery system consists of following components: the active targeting peptide RGD, an inorganic calcium phosphate (CaP) shell and an organic inner core. VRP and NVT were loaded into CaP shell and phosphatidylserine polyethylene glycol (PS-PEG) core of nanoparticles (NPs) separately to obtain NVT- and VRP-loaded NPs (NV@CaP-RGD). These codelivered NPs allowed VRP to prevent the efflux of NVT from breast cancer cells by competitively combining with drug efflux pumps. Additionally, NV@CaP-RGD was effectively internalized into breast cancer cells by precise delivery through the effects of the active targeting peptides RGD and EPR. The pH-triggered profile of CaP was also able to assist the NPs to successfully escape from lysosomes, leading to a greatly increased effective intracellular drug concentration.

**Conclusion:**

The concurrent administration of VRP and NVT by organic/inorganic NPs is a promising therapeutic approach to reverse MDR in breast cancer.
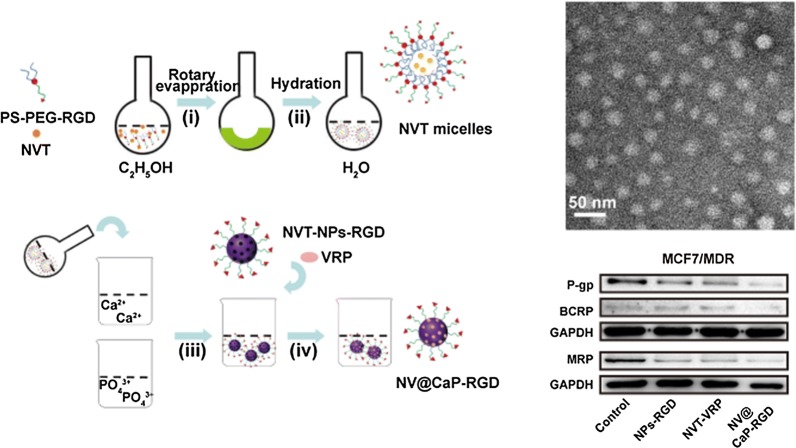

## Background

The incidence of breast cancer has increased substantially in recent decades [1, 2]. It is the main reason of cancer-related mortalities in women. Chemotherapy is an important part of the comprehensive treatment of breast cancer [3]. However, some breast tumors might be intrinsically resistant to chemotherapy or acquire multidrug resistance (MDR) during treatment [4, 5]. The present treatment of MDR breast cancer is multimodal, involving surgery, radiotherapy, combined chemotherapy, etc. Due to the high risk of early recurrence within 1–3 years after surgery and radiotherapy, advanced MDR breast cancer patients still prefer combined chemotherapy for its synergistic effect [6, 7].

The most common cause of MDR is a deficient intracellular concentration of chemotherapeutic drugs that present weak lethality to breast cancer cells and strong side effects [4, 5, 8, 9]. As the primary active drug efflux proteins, the ATP-binding cassette (ABC) transporter family, including P-glycoprotein (P-gp), multidrug resistance-associated protein (MRP) and breast cancer resistance protein (BCRP), utilizes the energy provided by ATP to pump drugs out of tumor cells to protect tumor tissue from chemical toxicity, leading to an insufficient intracellular concentration and a poor therapeutic efficiency of drugs [10, 11]. On the other hand, the insufficient cellular uptake of drugs, an acidic environment and the enzymes in lysosomes show great potential to decrease the intracellular concentration of drugs [12, 13]. Hence, an improvement in the intracellular concentration of chemotherapeutic agents is urgently needed.

Recently, there was great progress in the application of nanotechnology for treating malignant breast cancer [14], because nanoparticles (NPs) can actively or passively target tumor cells to improve the intracellular drug concentration, enhance the curative effect and reduce systemic toxicity [15]. In addition, micelle NPs with surface modifications exhibited pH sensitivity to improve the ability to escape from lysosome [16]. However, less work was conducted to combine two kinds of drugs in one NP to generate synergistic effect, or a successfully escape from the lysosomes to increase the effective intracellular drug concentration. Herein, a drug platform that synergistically delivers different drugs into breast cancer cells following successful lysosome escape is urgently required to improve the curative effect and alleviate the side effects.

In this study, based on the mechanism of MDR in breast cancer cells and the advantages of micelle NPs, we designed a new drug carrier system, RGD-PS-PEG calcium phosphate nanoparticles (NV@CaP-RGD) to incorporate novantrone (NVT) and verapamil (VRP) by a bio-mineralization method, where an organic phosphatidylserine polyethylene glycol (PS-PEG) micelle core encapsulates the chemotherapeutic agent NVT, an inorganic calcium phosphate (CaP) shell adsorbs the MDR inhibitor VRP, and a peptide RGD (H-(D-Val)-Arg-Gly-Asp-Glu-OH) acts as active tumor-targeted molecule to precisely guide the NPs to tumor cells [17, 18]. VRP was usually used as a drug efflux inhibitor by competitively binding to drug efflux pumps, and further degrading the function of drug efflux [19–21]. NVT, a broad-spectrum antitumor drug, is widely used for breast cancer treatment through intercalation with DNA strands and inhibition of the activity of topoisomerase II (Topo II) [22, 23]. The block copolymers PEG-PS (polyethylene glycol-phosphatidylserine) can self-assemble into anionic micelles in aqueous solution. The exposed PEG hydrophilic chain can form a hydrated film to prevent the aggregation of CaP and effectively enhance the stability of the NPs, help the nanoparticles (NPs) escape phagocytose of the reticuloendothelial system, and prevent the drugs leak from NPs during circulation [24–27]. CaP is a biocompatible, easily synthesized and degradable inorganic biomaterial, adsorbing on the surface of the NPs through loose electrostatic interactions and condensing VRP on the shell of NPs. It is worth noting that CaP can rapidly dissipate from NPs under lysosomal acidic conditions followed by the formation of ion pairs with lysosomal membranes to burst the lysosomes and increase the concentration of effective drugs in tumor cells [28–30]. In addition, NPs can precisely guide drugs into cells through enhanced permeability and retention (EPR) effects and the high affinity of the tumor-targeting peptide RGD for the integrin *α*_*v*_*β*_*3*_ receptor, which is highly expressed in tumor angiogenic blood vessels and breast cancer cells but rarely expressed in the normal cells [31–33]. Therefore, this novel drug delivery carrier greatly improved the intracellular concentration of drugs by inhibiting drug efflux, escaping from the lysosome, targeting tumors, reversing the MDR and enhancing the curative effect on breast cancer.

In this work, we also investigated whether NV@CaP-RGD can effectively promote the apoptosis of MDR breast cancer cells by inducing mitochondrial apoptosis and inhibiting the extracellular regulated protein kinases (ERK) signaling transduction pathway to achieve the best inhibitory effect on breast cancer cells in vitro [34–37]. Additionally, the antitumor activity was evaluated in vivo with the BALB/c subcutaneous MDR breast cancer xenograft model, suggesting that NV@CaP-RGD not only improved anti-cancer efficacy but also reduced side effects, depending on the profile of tumor targeting, long circulation and escape from the reticuloendothelial system. Consequently, NV@CaP-RGD provides a beneficial therapeutic approach to reverse MDR in breast tumor cells.

## Experimental section

### Materials, cell lines, and animals

Phosphatidylserine (PS) was purchased from Shanghai Yiji Medicine & Chemical Co, Ltd. Boc-NH-PEG-COO-NHS, Poly-(ethylene glycol) methyl ether (PEG, Mn = 2000), Rb (rhodamine b) and CaCl_2_ were obtained from Sigma-Aldrich, and RGD was purchased from Corner Stone Therapeutics (Shanghai), Ltd (Shanghai, China).

VRP was purchased from Melonepharma Co, Ltd, Dalian. NVT was purchased from Chongqing Carelife Pharmaceutical Co, Ltd. (NH_4_)_2_HPO_4_ was obtained from Sinopharm Chemical Reagent Co., Ltd.

MCF7/MDR cells (MDR human breast cancer cells) and MCF7 cells were obtained from the Institute of Biochemistry and Cell Biology at the Chinese Academy of Sciences (Shanghai, China). BALB/c nude mice were provided from the Animal Experiment Centre of Shanghai Cancer Institute.

### Preparation of co-delivery nanoparticles (NV@CaP-RGD)

NV@CaP-RGD was prepared by the bio-mineralization method. Briefly, NVT and PS-PEG-RGD was rotary evaporated to form a drug film, which was hydrated to form NVT micelle solution through CaCl_2_ solution. Then, (NH_4_)_2_HPO_4_ solutions were dropped into the NVT micelle solution. The centrifugation-redispersion cycles washed the white reaction products. Next, this acquired dispersion system and VRP were mixed by an ultrasonic processor. After stabilizing and further sonicated, NV@CaP-RGD were harvested by centrifugation and freeze-dried. The EE% of VRP or NVT in NPs was detected using high performance liquid chromatography (HPLC) assay after ultracentrifugation. Release profile of VRP and NVT from NPs in vitro was detected by a dialysis method (Millipore, USA) and determined using HPLC.

### ATP-consuming assays and Calcein AM assay

The ATP-consuming of NPs and NPs-RGD was measured through an ATP Assay Kit in MCF7 and MCF7/MDR cells. The Calcein AM assay of VRP-NVT, NV@CaP or NV@CaP-RGD were measured using fluorescein Calcein AM (Sigma-Aldrich) by a microplate reader. The % relative fluorescence was expressed as:

$$ \% {\text{Relative}}\;{\text{Fluorescence}}\;\left( {{\text{FL}}} \right) = \left[ {\left( {{\text{FLtreatment}}{-}{\text{FLnontreatment}}} \right)/{\text{FLnontreatment}}} \right] \times 100\% . $$

### Cellular uptake assay

The cellular uptake of NPs-RGD was assayed using fluorescence microscopy for direct images and HPLC machine for accurate internalization. HPLC system was used to analyze the drug concentration and the BCA protein kit was used to correct the test results. LysoTracker red probe and DAPI (4′,6-diamidino-2-phenylindole) were used to detect the lysosomal escaping using an FV-1200 Olympus confocal microscope.

### Detection of treatment effect in vitro

Annexin V-PI with flow cytometry analysis was used to investigate tumor cells apoptosis. JC-1 probe was performed to detect the mitochondrial membrane potential. 3-(4,5-Dimethyl-2-thiazolyl)-2,5-diphenyl-2-*H*-tetrazolium bromide (MTT) assays were used to detect the cell toxicity.

### Live imaging for biodistribution

After intravenous injection of Dir-NPs, Dir-NPs-RGD and unbounded Dir (1,1-dioctadecyl-3,3,3,3-tetramethylindotricarbocyaine iodide), the fluorescence images of nude mice were observed using in vivo imaging apparatus (LB983, Berthold Technologies Gmbh & Co. KG, Bad Wildbad, Germany). After experiment, the harvested normal organs (heart, spleen, liver, lung, kidney and tumor) were observed. The quantitative data was analyzed by *indiGo* software.

### Tumor growth inhibition study in vivo

The mice were treated with PBS solution, unbounded NVT and VRP, NV@CaP, and NV@CaP-RGD through intravenous injection at the dose of VRP 2 mg kg^−1^ and NVT 2 mg kg^−1^. The tumor size and body weigh were measured every 2 days. After final treatment, all tumors were collected for hematoxylin–eosin (H&E) staining and TUNNEL (terminal deoxynucleotidyl transferase dUTP nick end labeling) staining.

### Safety evaluation

After the final treatment, serum was collected to detect liver and kidney function. The heart, spleen, liver, lung, kidney and tumor were collected to evaluate toxicity of drugs through H&E staining.

### Statistical analysis

All statistical analyses were performed using SPSS version 21.0. The experimental data were statistically analyzed by using the t-test between independent samples. P values were considered statistically significant when less than 0.05.

## Results and discussion

### Tumor-targeted codelivery of NV@CaP-RGD nanoparticles with suitable size

To realize incorporation of the MDR inhibitor VRP and the chemotherapeutic agent NVT to treat MDR breast cancer, an organic/inorganic drug delivery system was created to encapsulate these two agents.

**Fig. 1:**
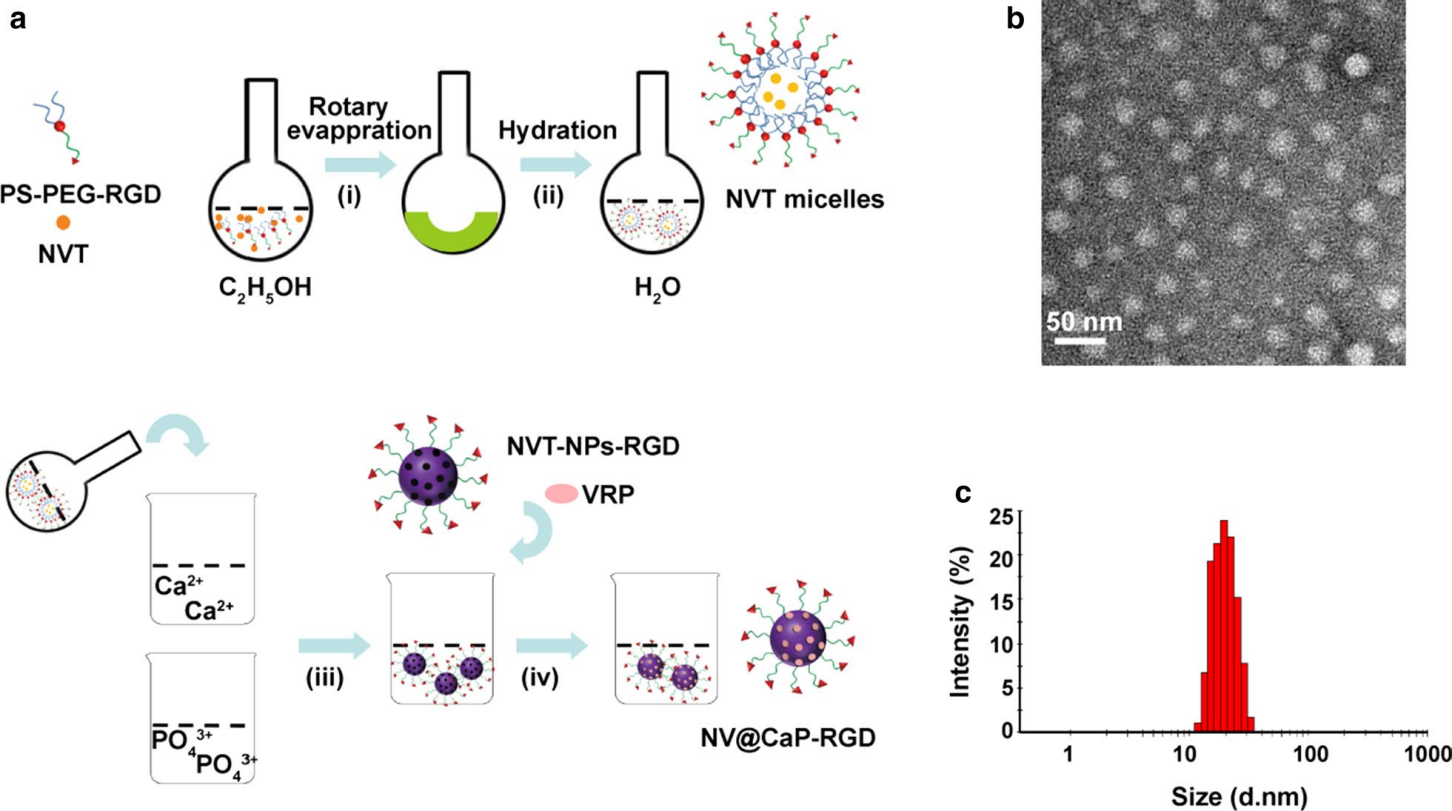
The preparation route of the suitably-sized nanoparticles NV@CaP-RGD. **a** The schematic route of NV@CaP-RGD. (i) PS-PEG-RGD and NVT was rotary evaporated to obtain drug film. (ii) The drug film self-assembled to the NVT micelles. (iii) (NH_4_)_2_HPO_4_ solutions were dropped into the NVT micelle solution. (iv) VRP was adsorbed into the micropores and condensed on the surface of the NPs through the calcium ions and phosphate ions (CaP) to obtain NVT- and VRP-loaded nanoparticles (NV@CaP-RGD). **b** The NV@CaP-RGD were spherical and no obvious aggregation. **c** The mean hydrodynamic diameter of NV@CaP-RGD was 29.82 ± 7.16 nm

Briefly, the carrier material (PS-PEG-RGD) was synthesized by condensation of the amine group from PS and the carboxyl group from PEG-COOH to form PEG-PS, followed by successfully conjugation of the RGD peptide to obtain PS-PEG-RGD [38]. In this compound, the PS-PEG self-assembled into anionic micelles in water owing to the PS hydrophobic moiety and the PEG hydrophilic moiety, and the RGD peptide was advantageous for active targeting to the tumor tissue. Then, the organic/inorganic codelivery nanoparticles NV@CaP-RGD were prepared by the biomineralization method (Fig. 1a) [38–41], in which the chemotherapeutic agent NVT was encapsulated into the PS-PEG-RGD micelles and the MDR inhibition agent VRP was adsorbed into the CaP shell. NV@CaP-RGD exhibited satisfactory compatibility (PS is the cell membrane component, PEG and RGD are proven drug carriers, and CaP is biodegradable) [42] and showed good dispersity from PEG through the creation of steric hindrance on the surface of the NPs, possessing a long circulatory property by PEG avoiding phagocytosis by the reticuloendothelial system as well as protecting VRP and NVT from degradation [24–26, 43–45].

NV@CaP-RGD was rough spherical particles without conjugation as detected by transmission electron microscopy (TEM) (Fig. 1b). Moreover, Fig. 1c showed that the hydrodynamic diameter of the NV@CaP-RGD was 29.82 ± 7.16 nm and the polydispersity index (PDI) was 0.175, which contributed to the increasing drug accumulation in tumor tissues via the EPR effect [46]. The NV@CaP-RGD in aqueous solution were negatively charged with a zeta potential of − 7.22 ± 0.39 mV (Additional file 1: Figure S1), which can effectively avoid the reaction with enzymes and proteins in the blood circulation, and improve the stability of NPs in the blood circulation through intravenous administration [47]. Furthermore, the drug encapsulation efficiency (EE%) and drug loading (DL%) of NV@CaP-RGD were detected by HPLC, where the EE% and DL% were 95.1% and 5.8% for NVT and 96.5% and 6.2% for VRP, respectively, suggesting excellent drug loading capacity.

The release profile of NV@CaP-RGD was detected by the dialysis bag method (Additional file 1: Figure S2). The accumulated released amount of VRP reached the maximum at 300 min at pH 5.5 from the free VRP solution, comparing to 600 min at pH 5.5 from the NPs, suggestive of sustained release property of the NPs. On the other hand, 83% VRP was released from NPs at pH 5.5 compared to only 48% VRP at pH 7.4 after 300 min, suggestive of strong pH depended releasing of VRP. Similar sustained release profile in nanoparticles and pH depended releasing ability was observed for NVT in NPs.

Generally, VRP and NVT were released simultaneously from the unbound drugs, while the release rate of VRP was significantly faster than that of NVT at pH 5.5 and 7.4, suggesting that VRP was released from the NPs prior to NVT, which is positively correlated with the inhibitory effect on MDR breast cancer cells (Additional file 1: Figure S3). In this drug delivery system, VRP was adsorbed on the CaP shell of the NPs with a loose interaction, compared with the inner tightly encapsulated NVT. Due to the pH-sensitive characteristic of CaP, the CaP shell rapidly dissociated in the acidic microenvironment and rapidly released VRP [48, 49], followed with the inner core gradually release via the erosion of the nanomaterial matrix and slow release NVT. Owing to the leading release, VRP had sufficient time to bind to the MDR proteins to inhibit the efflux of the drugs prior to the release of NVT. Therefore, with the erosion of the nanomaterial matrix, NVT was gradually released and remained in the cells for a longer time to improve NVT’s therapeutic efficiency in MDR tumor cells.

Therefore, NV@CaP-RGD successfully incorporated drug efflux inhibitor VRP and chemotherapeutic agent NVT into an inorganic/organic platform with active targeting RGD peptide, suitable size, satisfactory drug encapsulation efficiency and sustained drug release, which would improve therapeutic effects on MDR tumor with minor side effect.

### Increased intracellular concentration of the chemotherapeutic agent in MDR breast cancer cells

The precondition of the antitumor effect of NV@CaP-RGD is that they can be effectively internalized and not pumped out of breast cancer cells. In our study, the active tumor-targeting peptide RGD can precisely guide the NPs to the breast cancer cells [32, 33]. In addition, the drug efflux inhibitor VRP effectively prevented the efflux of the chemotherapeutic agent from MDR cells [20, 21]. Thus, NV@CaP-RGD was able to significantly increase the intracellular drug concentration in MDR breast cancer cells.

**Fig. 2:**
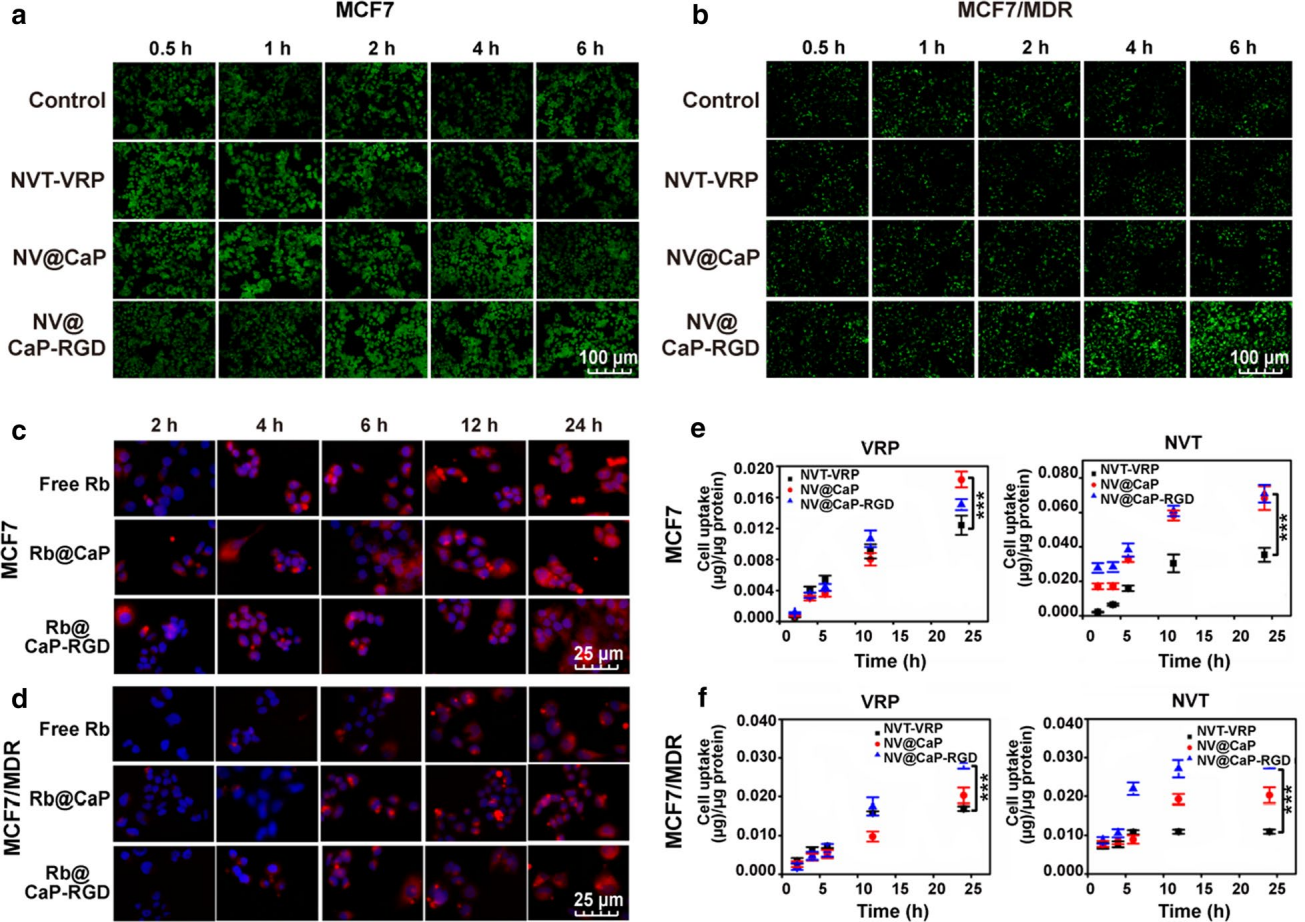
NV@CaP-RGD significantly increased the intracellular concentration of drugs in MCF7 and MCF7/MDR cells. **a**, **b** Fluorescence images of Calcein AM. The intracellular relative Calcein fluorescence in the NV@CaP-RGD group was significantly stronger than that in the control groups. As a substrate of efflux pump proteins, strong Calcein AM fluorescence indicates a significant inhibitory effect on the function of efflux proteins and more drugs remaining in the tumor cells. **c**, **d** The inverted fluorescence microscope technique assessed the internalization of the NPs. In MCF7 and MCF7/MDR cells, the amount of drugs loaded by NPs-RGD internalized by the cells was much greater than that of the unbound drugs and NPs. Over time, the amount of NPs internalized by the cells significantly increased. **e** and **f** The concentrations of VRP and NVT in MCF7 and MCF7/MDR cells were detected using HPLC. Compared to the NV@CaP-RGD and unbound VRP and NVT groups, NV@CaP-RGD substantially increased the intracellular concentrations of NVT and VRP (***P < 0.001)

Calcein AM, a substrate of efflux pump proteins, could be hydrolyzed into Calcein through an esterase to emit green fluorescence in live cells [50]. A Calcein AM assay was conducted to analyze the function of efflux pump proteins after MCF7 cells and MCF7/MDR cells were treated with unbound NVT-VRP, NV@CaP and NV@CaP-RGD (Additional file 1: Figure S4). The fluorescence intensity of Calcein is highly negatively correlated with the functions of efflux pump proteins, and the high level of green Calcein fluorescence indicated that more Calcein AM was in the cells and that there was a lower level of efflux pump proteins on the cell membrane. In MCF7 cells, the relative Calcein green fluorescence in all treated groups was stronger than that of control group. In contrast, for MCF7/MDR cells, the fluorescence was almost invisible in the control and unbound NVT-VRP groups, while the relative Calcein green fluorescence values were significantly stronger in the NV@CaP and NV@CaP-RGD groups, especially in the NV@CaP-RGD group (Fig. 2a, b). These data suggested that NV@CaP-RGD had a significant inhibitory effect on efflux pump proteins, leading to fewer drugs being pumped out of the MDR tumor cells.

We assessed the ability of the cells to internalize the NPs by inverted fluorescence microscopy. For MCF7 and MCF7/MDR cells, Rb-NPs-RGD presented the strongest fluorescence intensity compared to the unbound drugs and drug-NPs groups at the same time point (Fig. 2c, d, Additional file 1: Figure S5), suggesting that RGD-NPs could greatly enhance the breast cancer cellular uptake of the drugs. In addition, the intracellular concentrations of VRP and NVT in MCF7 and MCF7/MDR cells were detected using HPLC (Fig. 2e, f). Compared to the unbound VRP-NVT, NP-RGD substantially increased the intracellular concentration of drugs.

Therefore, NV@CaP-RGD can effectively increase the concentration of chemotherapeutic drugs in the cells and improve the therapeutic effects on MDR tumor cells, depending on the improvement of drug internalization and prevention of the efflux of the chemotherapeutic agents.

### Lysosomal escape assisted NV@CaP-RGD smoothly into nucleus

As NV@CaP-RGD entered into the tumor cells, they were recognized as foreign bodies to be transported into lysosomes. NVT plays the cytotoxic role on the premise of the escape from the lysosomes and entry into the nucleus [13]. Thus, the escape from lysosomes is crucial.

**Fig. 3:**
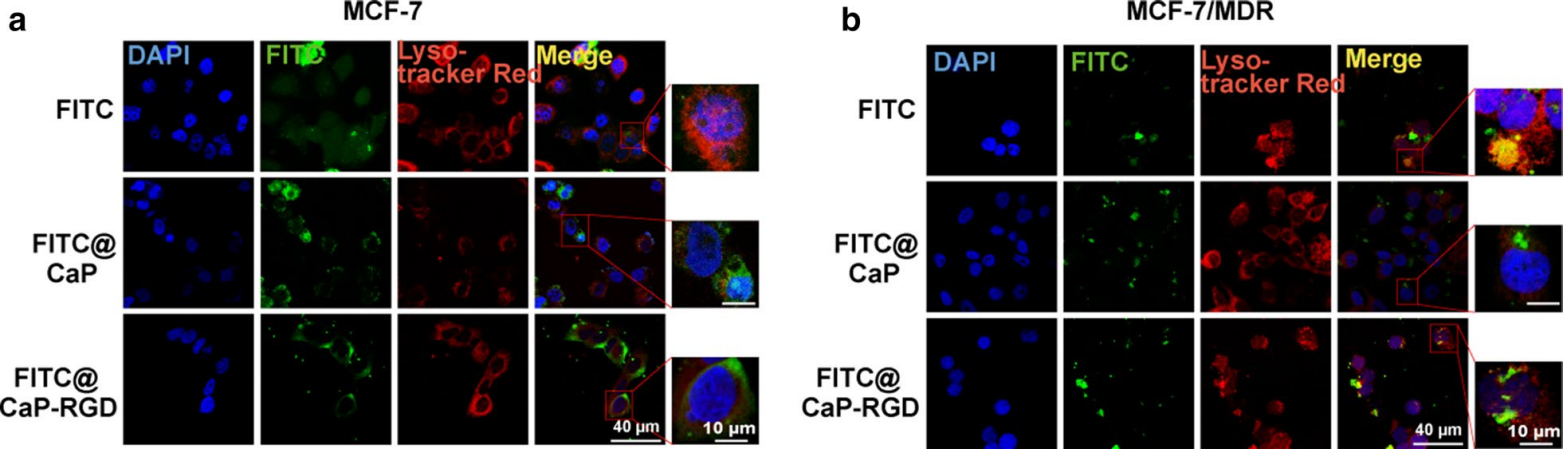
The lysosomal escape of NPs in **a** MCF7 cells and **b** MCF7/MDR breast cancer cells. In MCF7 and MCF7/MDR cells, the yellow fluorescence was very strong in the unbound VRP-FITC groups, suggesting that unbound VRP-FITC remained in the lysosomes. However, the yellow fluorescence became weaker or even disappeared in the RGD-NPs group, suggesting that the RGD-NPs had successfully escaped from the lysosomes. The NPs gave green fluorescence, lysosomes gave red fluorescence, and the nucleus gave blue fluorescence. The presence of the yellow color was due to the overlap of green and red fluorescence, suggesting that the NPs had entered the lysosomes

As shown in Fig. 3a, b, we observed by fluorescence microscopy that the NPs gave green fluorescence, the lysosomes gave red fluorescence, and the nucleus gave blue fluorescence. The presence of yellow fluorescence is due to the overlap of green fluorescence and red fluorescence, suggesting that the NPs had entered the lysosome. In MCF7/MDR cells, the yellow fluorescence was very strong in the unbound VRP-FITC group, suggesting that the drugs remained in the lysosomes. However, the yellow fluorescence became weak or even disappeared in the RGD-NPs group, suggesting that RGD-NPs had successfully escaped from lysosomes.

The CaP of the NPs is susceptible to digestion under the acidic conditions in the lysosomes (the pH value is approximately 5.5) [48, 49] along with effective bursting of the lysosomes through the formation of ion pairs with lysosomal membranes. Therefore, NV@CaP-RGD can effectively increase the concentration of the drugs in the nucleus and improve the therapeutic effect on MDR tumor cells through lysosomal escape.

### Enhanced induction of apoptosis and improved therapeutic effects in MDR breast cancer cells by NV@CaP-RGD

The DNA damage induced by NVT in cells could effectively promote apoptosis and the death of tumor cells [51]. Therefore, we investigated the therapeutic efficiency of NV@CaP-RGD in breast cancer cells via apoptosis experiments and cellular toxicity tests.

**Fig. 4:**
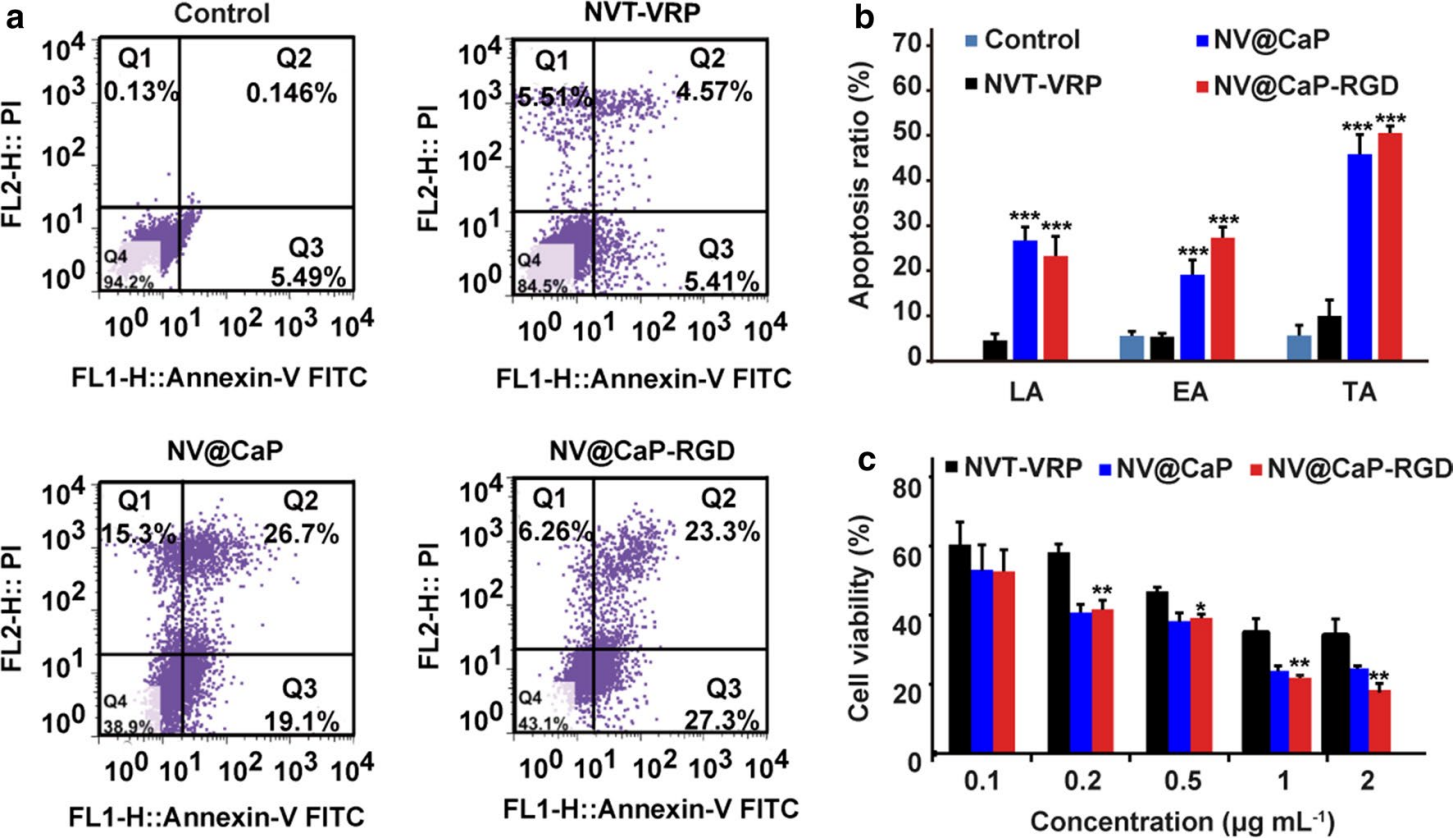
NV@CaP-RGD substantially promoted apoptosis and improved the therapeutic effect on MCF7/MDR cells. **a** An annexin V/PI double-staining assay was used to evaluate the apoptotic ratio of MCF7/MDR cells treated with unbound VRP-NVT, NV@CaP-RGD and NV@CaP for 72 h. **b** Quantitative analysis of the apoptosis ratio. Compared to unbound VRP-NVT, NV@CaP-RGD and NV@CaP were better able to induce apoptosis of MCF7/MDR cells, including early and late apoptosis, but no significant difference was found between these two groups (***P < 0.001 compared with the control group). The codelivery of VRP and NVT by NPs had a synergistic effect on the induction of apoptosis in MCF7/MDR cells. EA indicates early apoptosis, LA indicates late apoptosis, and TA indicates total apoptosis. **c** Cell viability of MCF7/MDR cells treated with various concentrations of unbound VRP-NVT, NV@CaP and NV@CaP-RGD for 72 h (***P < 0.001, **P < 0.01, *P < 0.05, compared with the control group). The cell viability significantly decreased in the NV@CaP-RGD and NV@CaP groups, suggesting that NV@CaP-RGD was able to effectively inhibit the viability of MDR breast cancer cells at the cellular level

Because apoptosis is a form of cell death, the effective induction of apoptosis by NV@CaP-RGD can obviously improve the therapeutic effect on MDR breast cells. The annexin V/PI double-staining assay was used to evaluate the apoptosis rate of MCF7 cells (Additional file 1: Figure S6A and B) and MCF7/MDR tumor cells (Fig. 4a, b) treated with unbound VRP-NVT, NV@CaP and NV@CaP-RGD for 72 h. The results showed that unbound VRP-NVT could induce the apoptosis of MCF7 and MCF7/MDR tumor cells to some extent. However, the apoptotic ratios of the NV@CaP and NV@CaP-RGD groups were significantly higher than that of the unbound VRP-NVT group, including early apoptosis and late apoptosis. Compared to the NV@CaP group, codelivery of VRP and NVT by RGD-NPs had a better synergistic effect on the induction of apoptosis in MCF7 and MCF7/MDR cells, depending on the active targeting effect of the RGD peptide.

To evaluate the effect of NV@CaP-RGD on the inhibition of MDR breast cancer cells, we used the MTT method to test the cell viability of MCF7 and MCF7/MDR cells incubated with unbound VRP-NVT, NV@CaP and NV@CaP-RGD at different concentrations (0.1, 0.2, 0.5, 1 and 2 μg mL^−1^) for 72 h. Compared to the control and unbound VRP-NVT groups, the viability of the MCF7 (Additional file 1: Figure S6C) and MCF7/MDR cells (Fig. 4c) was significantly lower in the NV@CaP and NV@CaP-RGD groups. Although unbounded VRP-NVT had certain inhibitory effect on MCF cells, while had a poor inhibitory effect and could not effectively kill MDR tumor cells. In contrast, NV@CaP-RGD was able to significantly promote apoptosis of the MDR breast cancer cells, resulting in powerful inhibition of the viability of MDR tumor cells at the cellular level.

### The apoptosis mechanisms of MDR breast cancer cells induced by NV@CaP-RGD

The mechanisms of the apoptosis route are complicated. Recently, the mitochondrial pathway has received increasing interest regarding its critical role in the transduction of apoptosis [34, 35]. In addition, the apoptosis-related protein extracellular signal-regulated kinase (ERK) and phospho-ERK (p-ERK), the key proteins in the Ras/Raf/MEK/ERK signaling pathway, adjust the activation of other factors to inhibit the apoptosis of the tumor cells [52–54]. Thus, enhancing mitochondrial apoptosis and inhibiting the function of the ERK signaling pathway can significantly promote the apoptosis of tumor cells.

**Fig. 5:**
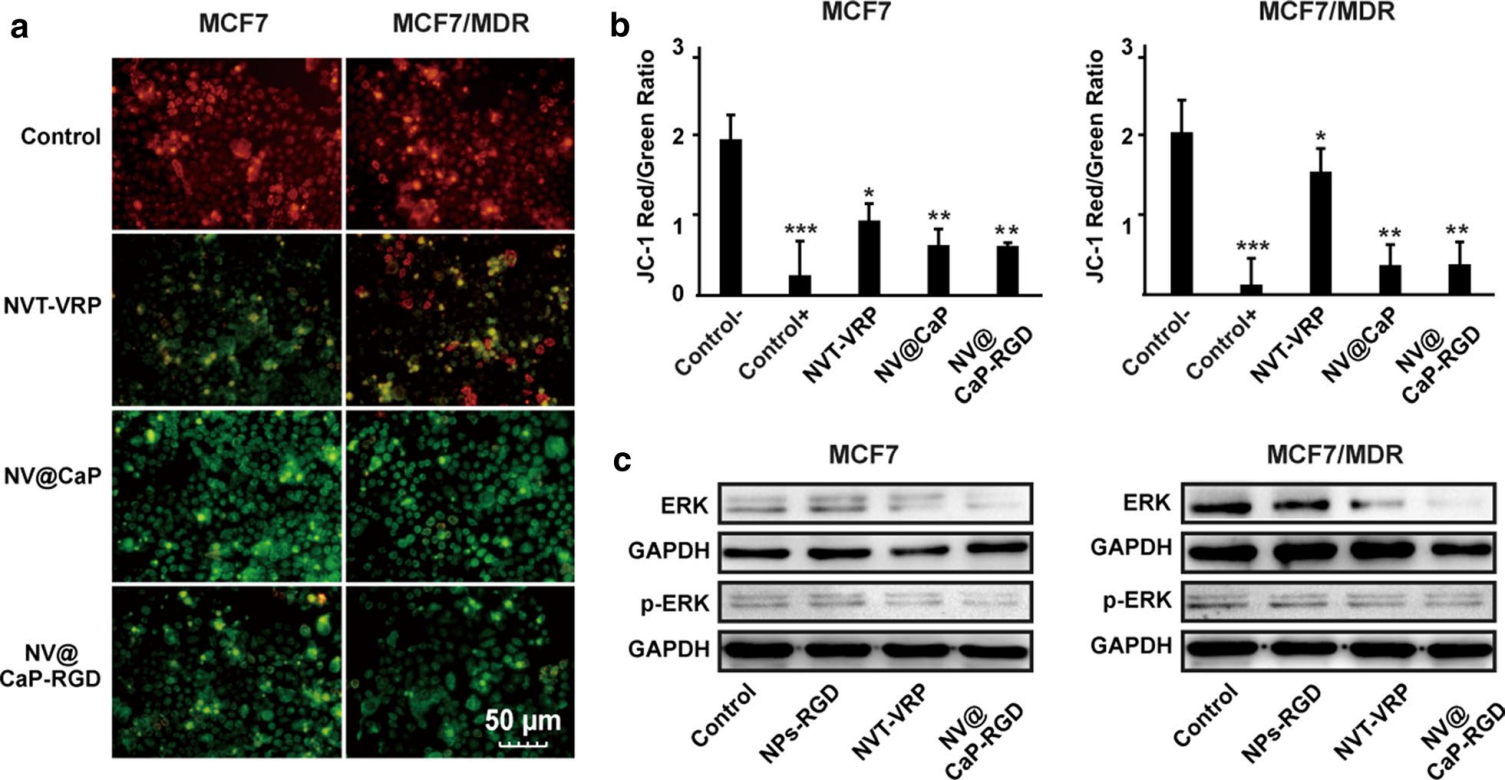
The mechanisms of apoptosis in MCF7 and MCF7/MDR cells induced by NV@CaP-RGD. **a** We used JC-1 to mark the mitochondrial membrane potential and fluorescence microscopy and flow cytometry to measure that potential. The green fluorescence in the NV@CaP-RGD and NV@CaP groups increased significantly in MCF7 and MCF7/MDR tumor cells. **b** The mitochondrial membrane potential of the MCF7 cells and MCF7/MDR cells decreased significantly when treated with NV@CaP-RGD and NV@CaP, and there was no significant difference between these two groups. Control+ : carbonylcyanide-m-chlorophenylhydrazone, Control−: nontreated group (***P < 0.001, **P < 0.01, *P < 0.05 compared with the nontreated group). **c** The expression of apoptosis-related proteins ERK and p-ERK. Significant downregulation of the proteins ERK and p-ERK was detected by Western blotting analysis, suggesting that NV@CaP-RGD significantly induced apoptosis in MDR breast cancer cells via inhibition of the ERK signaling transduction pathway. According to the above results, NV@CaP-RGD promoted apoptosis in MDR breast cancer cells by decreasing the mitochondrial membrane potential and inhibiting ERK phosphorylation

The decrease of mitochondrial membrane potential (MMP) is significant characteristic of mitochondrial apoptosis, reflecting the mitochondria apoptosis ratio [55, 56]. As an ideal fluorescent probe, J-aggregates (JC-1) have been widely used to detect mitochondrial membrane potential. The high mitochondrial membrane potential caused polymerized JC-1 to accumulate in mitochondrial matrix and produce red fluorescence; while JC-1 with the low mitochondrial membrane did not accumulate in the mitochondrial matrix, producing green fluorescence. As shown in Fig. 5a, b, MCF7 and MCF7/MDR cells were treated with unbound VRP-NVT, NV@CaP and NV@CaP-RGD for 72 h. In MCF7 tumor cells, compared to control group, the green fluorescence of all three experimental groups increased significantly. In contrast, in MCF7/MDR tumor cells, the green fluorescence intensity was very weak in the unbound VRP-NVT group while the green fluorescence of the NV@CaP-RGD group significantly increased, suggesting that NV@CaP-RGD effectively induced apoptosis in MDR breast cancer cells through the mitochondrial pathway.

We used western blotting to detect the expression of ERK and p-ERK in each treatment group in MCF7 and MCF7/MDR cells (Fig. 5c and Additional file 1: Figure S7). Compared to the control and unbound NVT-VRP groups, the expression of ERK and p-ERK was significantly lower in NV@CaP-RGD group, suggesting that NV@CaP-RGD significantly induced the apoptosis of MDR breast cancer cells via inhibition of the phosphorylation of ERK and interference of the ERK signaling transduction pathway.

In summary, NV@CaP-RGD can be effectively internalized into MDR tumor cells via optimized internalization pathways. After entering the tumor cells, the NPs are recognized as foreign bodies to be transported into lysosomes. The CaP from NV@CaP-RGD is susceptible to digestion, along with the formation of ion pairs with the lysosomal membranes to effectively burst lysosomes with notably increased intracellular drug concentrations. Due to the sequential release of the codelivered NPs, when the function of the efflux pump proteins is inhibited thoroughly by VRP (leading release), most NVT begins its gradual release and smoothly remains in tumor cells. Then, the increased amount of NVT in MDR tumor cells significantly improves cell apoptosis via the induction of mitochondrial apoptosis and inhibition of the ERK signaling transduction pathway, along with powerful inhibition of the viability of MDR breast cancer cells. Thus, NV@CaP-RGD is a promising tool for sensitizing MDR tumor cells to NVT.

### Functional inhibition of drug efflux in MDR breast cancer cells

The efflux of chemotherapeutic drugs is an important event for the MDR breast cancer. Due to the codelivery of NVT and VRP via NPs, the drug efflux inhibitor VRP was able to effectively inhibit the efflux of the chemotherapeutic agent NVT. In addition, NV@CaP-RGD can inhibit the function of efflux pump proteins by consuming a large amount of intracellular ATP [57, 58]. Through these two points, NV@CaP-RGD can effectively prevent the efflux of the chemotherapeutic agent NVT from MCF7/MDR cells.

**Fig. 6:**
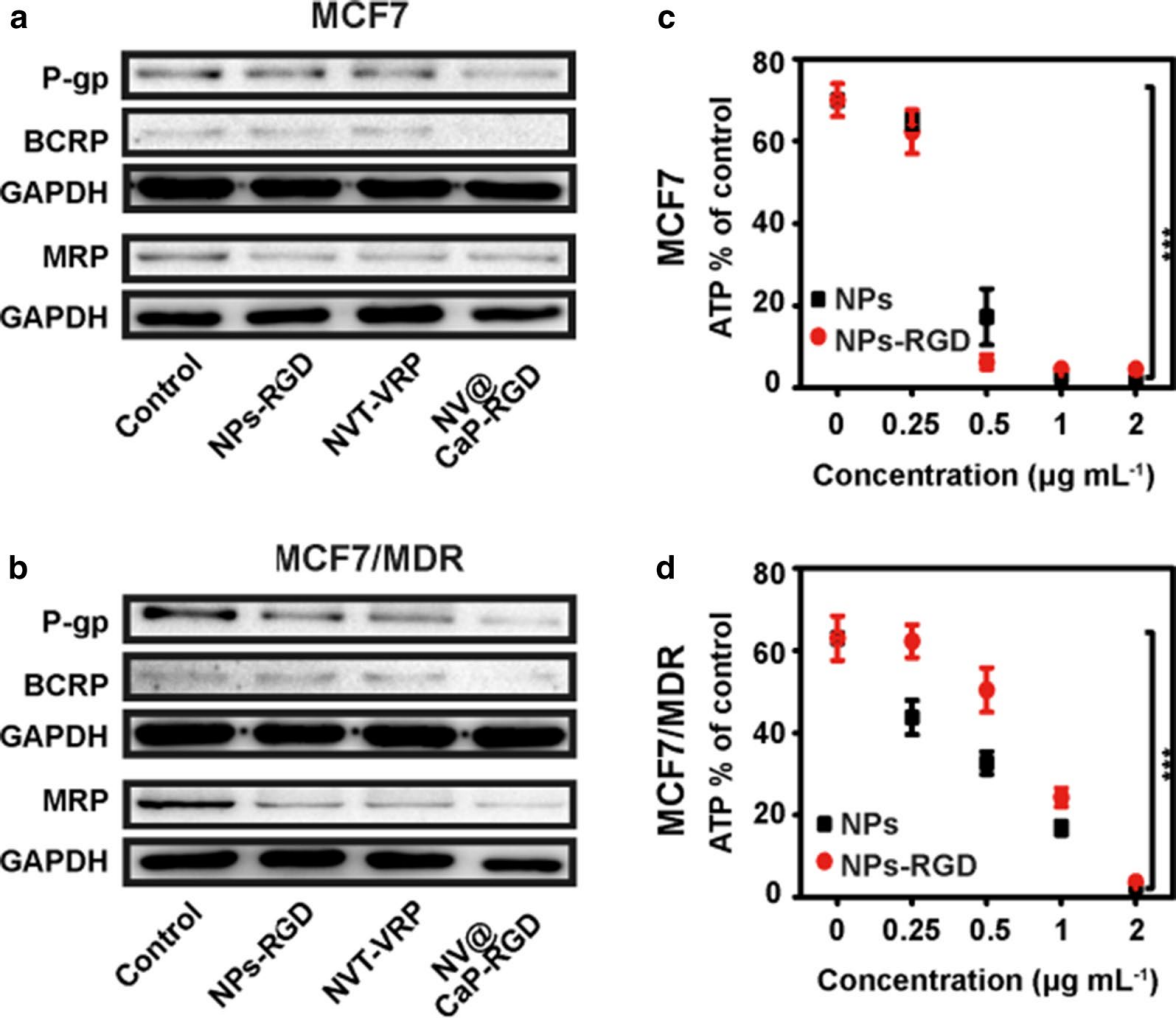
NV@CaP-RGD effectively inhibited the expression of drug efflux proteins and decreased the intracellular concentration of ATP in MDR breast cancer cells. Western blotting images of the expression of efflux pump proteins in **a** MCF7 cells and **b** MCF7/MDR cells. Western blotting analysis showed that the expression of efflux pump proteins (P-gp, MRP and BCRP proteins) significantly decreased in the NV@CaP-RGD group. **c**, **d** The amount of ATP in MCF7 and MCF/MDR tumor cells treated with various concentrations of RGD-NPs and NPs. As the concentration increased, the ATP concentration decreased substantially (***P < 0.001 compared with the nontreated control group). Due to insufficient energy, the function of the efflux pump proteins was significantly inhibited

Efflux pump proteins (BCRP, MRP and P-gp) have been determined in several malignant tumors, including breast cancers [10, 11, 59, 60]. Western blotting was used to detect the expression of these proteins in breast cancer cells after treatment with NPs-RGD, unbound NVT-VRP and NV@CaP-RGD. As shown in Fig. 6a, b and Additional file 1: Figure S8, the proteins BCRP, MRP and P-gp were significantly downregulated in the NV@CaP-RGD group in both MCF7 and MCF7 MDR cells, especially P-gp.

Because the function of efflux pump proteins correlates with the amount of ATP in the cells, we detected the concentration of intracellular ATP after incubation with the various concentrations of NPs or NPs-RGD (Fig. 6c, d). As the concentration increased, the intracellular concentration of ATP was substantially decreased in MCF7 and MCF7/MDR breast cancer cells. Compared with the nontreated group, RGD-NPs and NPs could consume a lot of ATP in breast cancer cells (P < 0.001). As the primary active drug efflux proteins, the ABC transporter family, including P-gp, MRP and BCRP proteins, utilizes the energy provided by ATP hydrolysis to pump drugs out of tumor cells, leading to an insufficient intracellular concentration and a poor therapeutic efficiency of drugs [10, 11]. Therefore, Due to the energy deficiency, the function of the efflux pump proteins was significantly inhibited, and the efflux efficiency of the drugs was inhibited.

Efflux pump proteins are able to discharge chemotherapeutic drugs out of the tumor cells so that the sensitivity of the tumor cells to the chemotherapeutic drugs greatly decreases. In this study, NV@CaP-RGD could not be well pumped out of the cells via the inhibition of the function of the efflux pump-related proteins, the consumption of a large amount of intracellular ATP, and the increased intracellular concentration of drugs. Thus, NV@CaP-RGD provided an effective approach to prevent the efflux of drugs.

### The distribution of RGD-NPs in vivo

To achieve the anticipated therapeutic effects in vivo, it is essential that chemotherapeutic agents can accurately accumulate around the tumor and play a sustained role in killing the tumor cells. The NPs’ profile of tumor targeting and a long circulation time can successfully achieve the above two aims.

**Fig. 7:**
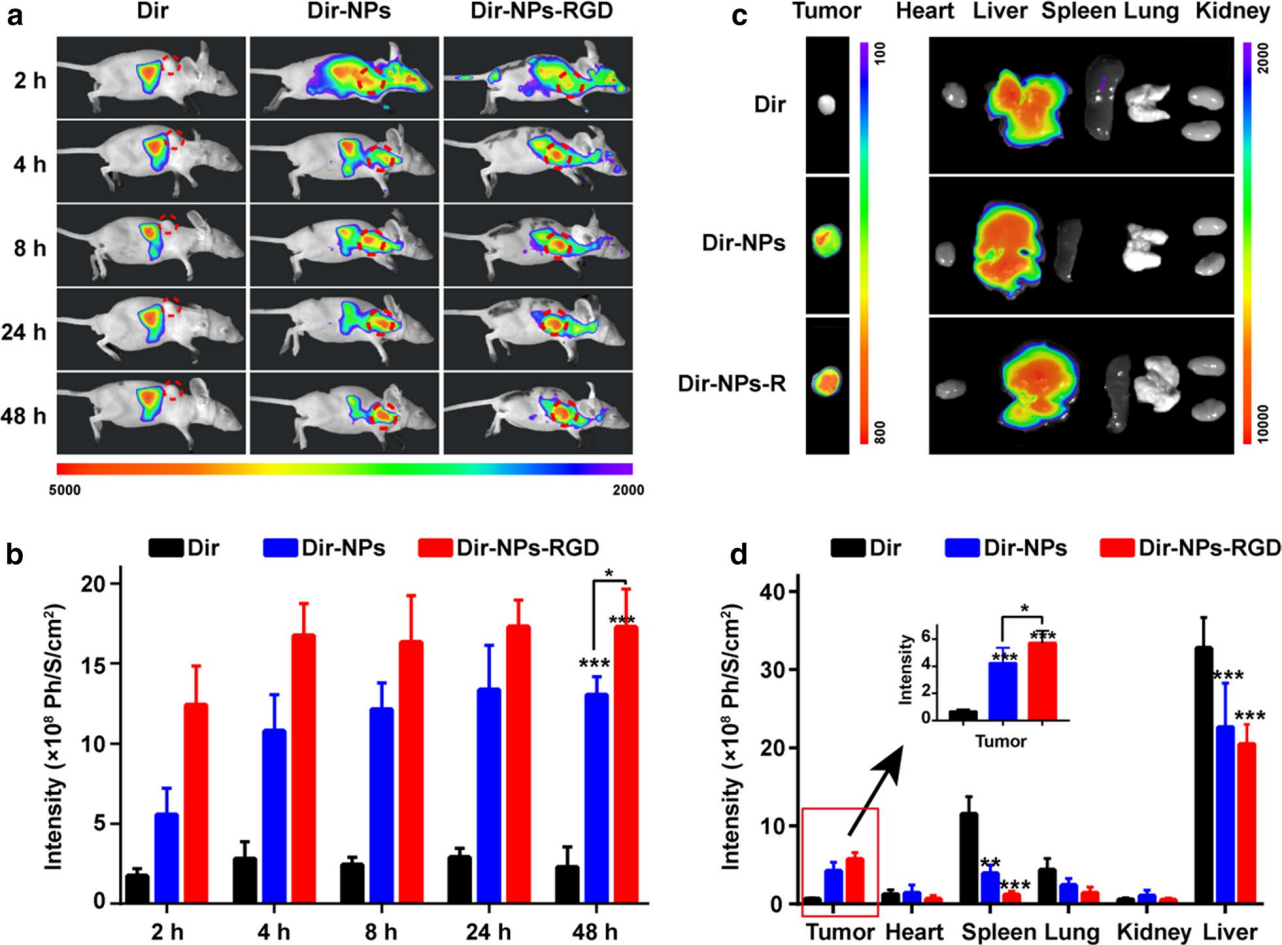
The distribution of codelivery NPs in vivo. **a** Fluorescence images after intravenous injection at various times. Compared with the main distribution of Dir in the abdominal cavity in the unbound Dir group, Dir-NPs and Dir-NPs-RGD were mainly distributed in MDR tumor tissues (red circle) and the abdominal cavity over time, suggesting better tumor targeting by RGD-NPs. **b** Quantitative average fluorescent intensities in MDR tumors after intravenous injection. *P < 0.05, **P < 0.01, ***P < 0.001, compared with unbound Dir group. **c** Ex vivo fluorescence images of the isolated MDR tumors, hearts, livers, spleens, lungs and kidneys after intravenous injection after 48 h. **d** The mean quantitative fluorescence intensity of the isolated organs in all groups. The fluorescence of Dir-NPs-RGD group in tumor was significantly stronger than that of the Dir-NPs and unbounded Dir groups, along with the relatively lower concentration of Dir in other normal tissues and organs

Dir were applied to live imaging in vivo to study the tumor targeting of the NPs (Fig. 7a). Initially, after injection of the unbound Dir, Dir-NPs and Dir-NPs-RGD into nude mice, unbound Dir was mainly distributed in the thoracic cavity and abdominal cavity, while Dir-NPs and Dir-NPs-RGD were equally distributed in the tumors and the abdominal cavity. Over time, the fluorescence intensity of the tumor tissue gradually increased, along with a gradual decrease of the fluorescence in the abdominal cavity. The fluorescence intensity of Dir-NPs-RGD in tumors was stronger than that of Dir-NPs, indicating better tumor targeting. At 48 h post injection, the fluorescent signal inside the tumor in the Dir-NPs and Dir-NPs-RGD groups was still observed (Fig. 7b), suggesting that the drugs encapsulated by NPs can stay in tumor tissues for a long time.

Forty-eight hours after injection, the biodistribution of Dir in the major organs and tumors was evaluated. The Fig. 7c, d showed that the fluorescence intensity of the Dir-NPs-RGD group in the tumors was obviously stronger than that of the Dir-NPs and unbound Dir groups, and there was a relatively lower concentration of Dir in the other normal tissues and organs. The fluorescence signals in the NPs group was weaker in the liver and spleen because most of the NPs were not captured by the mononuclear phagocyte system (MPS) or the reticuloendothelial system (RES) in the liver and spleen.

Thus, depending on the fact that NPs can accumulate in the tumor site accurately via the action of the tumor-targeting peptide RGD, the EPR effect, the escape from the reticuloendothelial system and a longer circulation time in vivo, NPs can not only help the drug achieve the best therapeutic effect but also effectively reduce the side effects on normal tissues and organs.

### Improving the curative effects on MDR breast cancer tumors by NV@CaP-RGD with minor side effects

To evaluate the tumor therapeutic efficiency of NV@CaP-RGD in vivo, the MCF7/MDR subcutaneous tumor xenografts in nude mice were subjected to various formulations including unbound NVT-VRP, NV@CaP and NV@CaP-RGD every 4 days for 4 weeks through intravenous injection. Tumor growth, TUNEL staining and H&E staining were used to assess the therapeutic efficiency.

**Fig. 8:**
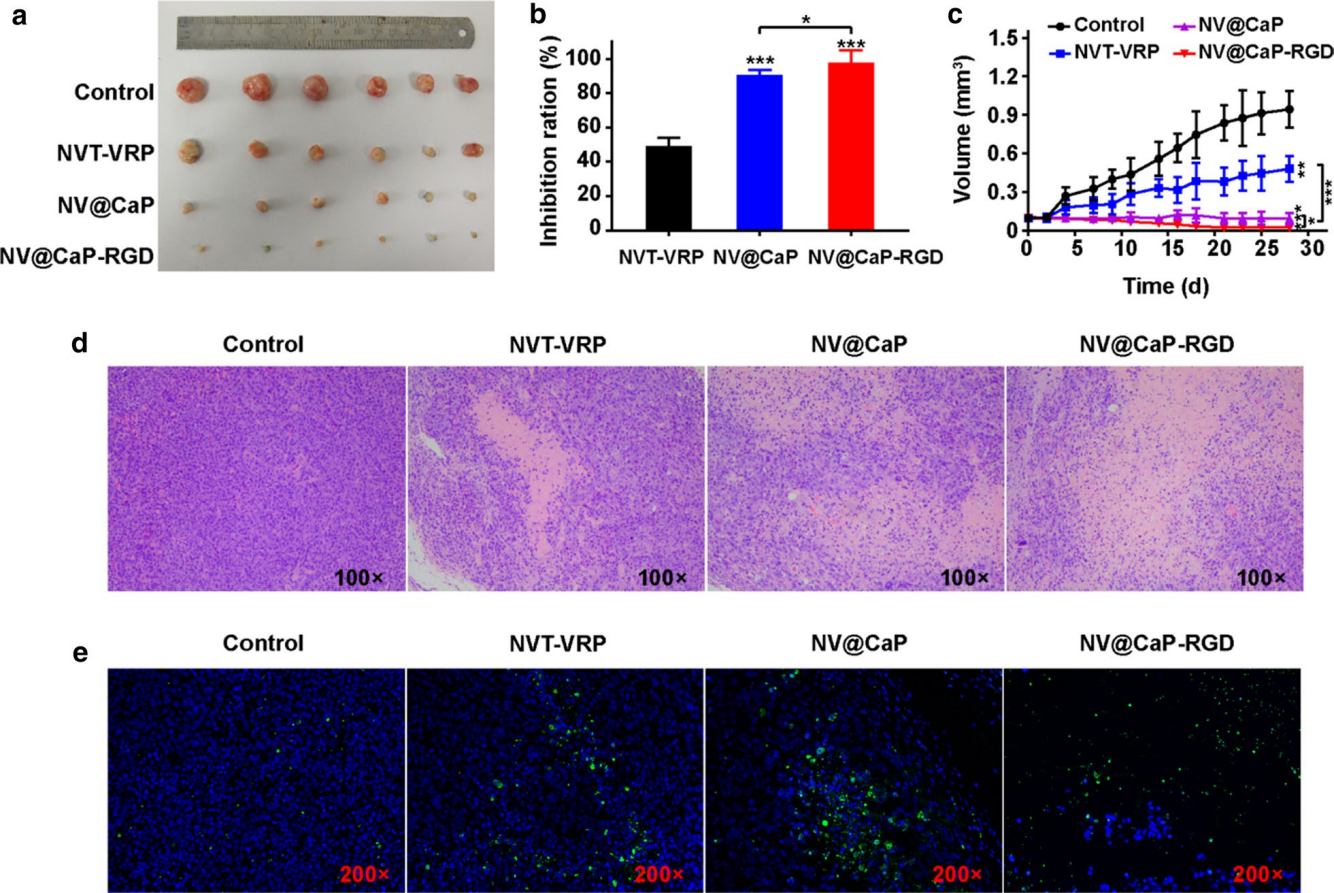
Significant curative effect on the MDR breast cancer tumor in vivo. **a** Images of MCF7/MDR subcutaneous xenograft tumors at the treatment endpoint. **b** Tumor inhibition ratio. Compared to the tumor inhibition ratio (49.7%) in the unbound NVT and VRP groups, the tumor inhibition ratios were obviously improved in the NV@CaP-RGD group (90.1%, P < 0.001) and NV@CaP group (83.3%, P < 0.001). In addition, compared to the NV@CaP group, NV@CaP-RGD achieved a better therapeutic effect on breast MDR tumors through the tumor-targeting peptide RGD (P < 0.05). *P < 0.05, **P < 0.01, ***P < 0.001, compared with unbound drugs group. **c** Tumor growth curves during the treatment period. The tumor volume of the NV@CaP-RGD treatment group was 22.71 ± 14.96 mm^3^ at the endpoint, which was the smallest one among all groups. **d** H&E images (×100) of the MDR breast cancer tumors. NV@CaP-RGD can promote tumor necrosis to enhance its therapeutic effects on MDR breast cancer. **e** TUNEL analysis (×200) of tumor tissues after treatment with various drug formulations. There was obvious apoptosis of MDR breast cancer cells in NV@CaP-RGD group, consistent with the previous results

After the treatment, the tumors were harvested (Fig. 8a). Compared to the tumor volume of 943.02 ± 141.4 mm^3^ in the control group, the tumors in the unbound NVT-VRP group had a size of 480.25 ± 101.79 mm^3^, indicating that the unbound NVT-VRP had some regressive effect on MDR tumors. However, the tumor volumes of the NV@CaP and NV@CaP-RGD groups were reduced to 91.03 ± 41.04 mm^3^ and 22.70 ± 14.96 mm^3^, respectively, which was much smaller than that of the unbound NVT-VRP group. NV@CaP achieved good antitumor effects on MDR breast tumors owing to the passive tumor targeting (EPR effect), a long circulation and reticuloendothelial system escaping ability. Furthermore, compared to NV@CaP, NV@CaP-RGD achieved a better curative effect on MDR breast tumors through the tumor-targeting peptide RGD (Fig. 8b, c). Histological changes and the apoptosis of tumor tissue, as evaluated by H&E staining (Fig. 8d) and TUNEL staining (Fig. 8e), showed that there was obvious cancer cell necrosis and apoptosis in the NV@CaP-RGD group, indicating that the administration of NV@CaP-RGD improved the sensitivity of breast cancer cells to NVT and effectively improved cell necrosis and apoptosis, leading to significant anti-tumor effect.

### Safety evaluation of NV@CaP-RGD

**Fig. 9:**
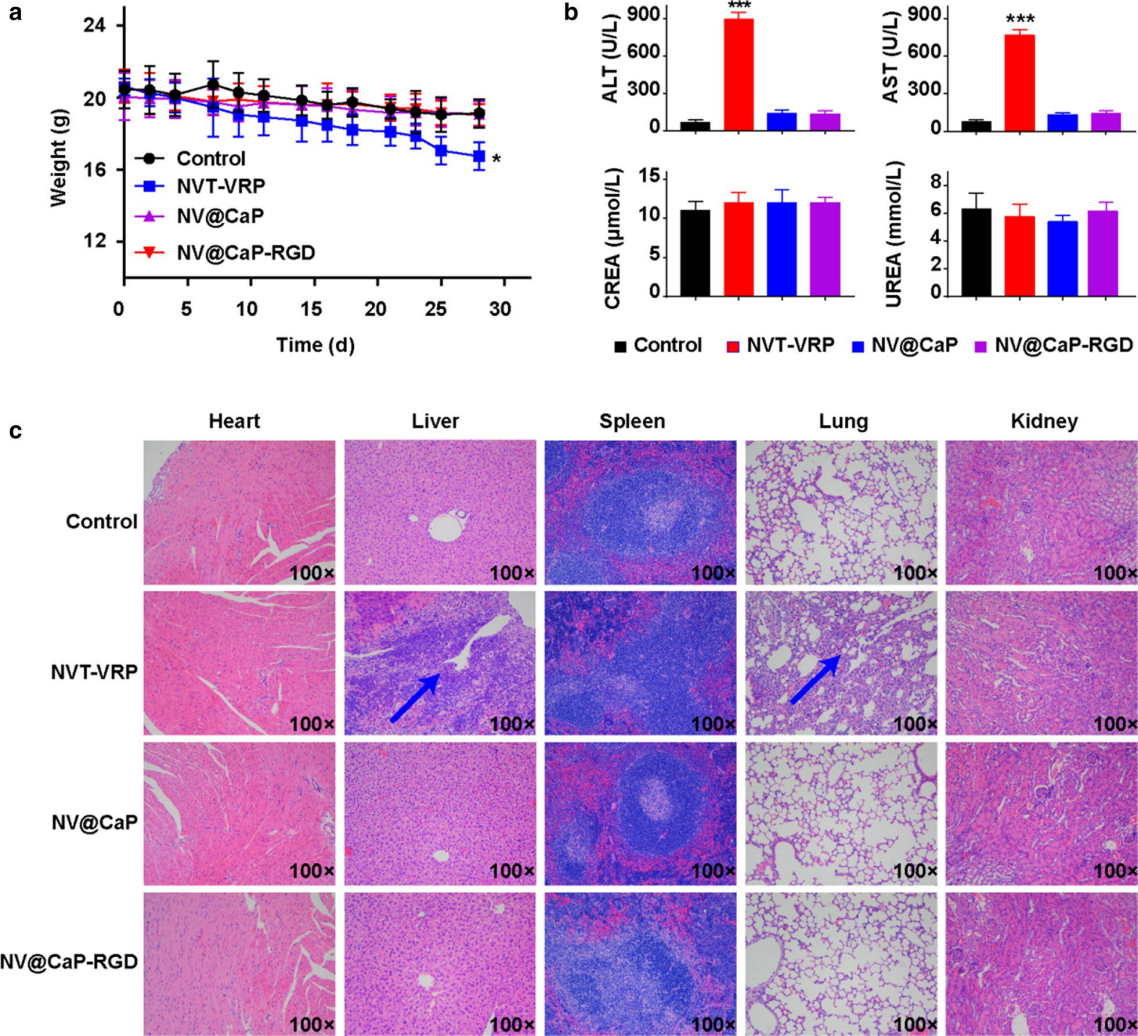
Minor toxicity for normal organs from NV@CaP-RGD in vivo. **a** Changes in the body weight of mice bearing MCF-7/MDR subcutaneous xenograft tumors over the treatment period. Compared to the NV@CaP-RGD and NV@CaP groups, the body weight of the mice in the unbound drug group slightly decreased during the entire experimental period (P < 0.05). **b** Indices of hepatic and renal function measured at 48 h after the final injection of different drug formulations in nude mice. The levels of ALT and AST in the unbound drug group were much higher than those in the other three groups (P < 0.001), suggesting obvious toxicity of unbound NVT and VRP in the liver. Compared to the untreated group, there were no significant changes in the levels of UREA and CREA in all of the treated groups, suggesting negligible nephrotoxicity. The *** stands for significant difference P < 0.001. **c** Histopathology of major organs from mice after intravenous injections of PBS and various drug formulations. Hepatic necrosis and a pulmonary inflammatory response were observed in the unbound NVT and VRP group without obvious pathological changes in other organs. No obvious pathological change in all organs was observed in the NV@CaP-RGD and NV@CaP groups, suggesting that the NPs can effectively reduce the toxicity of drugs to normal organs

To evaluate the safety of NV@CaP-RGD in vivo, weight loss, hepatic and renal function indices and H&E staining of the major organs were utilized to evaluate the toxicity. Obvious weight loss was observed in unbound NVT-VRP group, while the body weight barely declined after treatment with NV@CaP and NV@CaP-RGD in MCF7/MDR nude mice (Fig. 9a). The levels of aspartate transaminase (AST) and alanine transaminase (ALT) in the unbound NVT-VRP group were much higher than those in the other three groups (P < 0.001), suggesting that the NPs greatly alleviated the liver toxicity of the drugs (Fig. 9b). The levels of uremic (UREA) and creatinine (CREA) were not significantly different in all groups, indicating minor renal toxicity (Fig. 9b). Necrosis in the liver and an inflammatory response in the lungs were observed in the unbound NVT-VRP group in the H&E images while no obvious pathologic change was in the NV@CaP and NV@CaP-RGD groups (Fig. 9c), suggesting that the unbound NVT-VRP was harmful to the liver and lungs and that the NPs were able to reduce the toxicity of the drugs to normal organs.

In this work, the materials for preparing NPs were demonstrated to be low toxic; high drug encapsulation avoided burst release to reduce acute toxicity in vivo; tumor targeting could reduce drug concentration in normal tissues. Therefore, NV@CaP-RGD can effectively inhibit the proliferation of MDR breast tumors in vivo without obvious toxicity to normal organs.

## Conclusions

To reverse the MDR of breast cancer, we successfully prepared the inorganic/organic nanoparticles NV@CaP-RGD to incorporate the drug efflux inhibitor VRP and chemotherapeutic agent NVT, where the PS-PEG core encapsulates the NVT, the CaP shell adsorbs the VRP, and the RGD peptide acts as an active tumor-targeting ligand to precisely guide the NPs to the breast cancer cells. NV@CaP-RGD can effectively increase the concentration of chemotherapeutic drugs in MDR tumor cells by improving drug internalization, preventing the efflux of chemotherapeutic agents and escaping from the lysosome, thereby achieving a higher inhibitory effect, increasing the apoptosis rate via the activated mitochondrial pathway, suppressing the ERK signaling transduction pathway in vitro, and generating improved therapeutic effects. Furthermore, owing to the tumor-targeting profile, the long circulation time and reticuloendothelial system escape, NV@CaP-RGD was essential not only for improving the antitumor efficacy but also for reducing the side effects in normal organs in vivo. Consequently, NV@CaP-RGD is an effective method for the treatment of MDR breast cancer.

## Supplementary information

**Additional file 1.** Additional figures and experimental details. **Figure S1.** The average potential of NV@CaP-RGD was − 7.22 ± 0.39 mV. **Figure S2.** NV@CaP-RGD showed sustained-releasing and sequential-releasing pattern compared with that of unbounded NVT-VRP. **Figure S3.** The MCF7/MDR cells were incubated with VRP for various time (0.5 h, 1 h, 2 h, 3 h, 4 h) before adding NVT into it. **Figure S4.** The quantitative fluorescence intensity of Calcein AM at 6 h. **Figure S5.** The statistics of the internalization ability. **Figure S6.** The NV@CaP-RGD substantially promoted apoptosis and improved the therapeutic effect on MCF7 cells in vitro. **Figure S7.** The quantitative grayscale intensity of EKR and p-EKR proteins in MCF7 and MCF7/MDR cells. **Figure S8.** The quantitative grayscale intensity of efflux pumps proteins in MCF7 and MCF7/MDR cells.

## Data Availability

All data generated or analyzed during this study are included in this published article and its Additional file.

## Abbreviations

PS-PEG: phosphatidylserine polyethylene glycol
CaP: calcium and phosphate irons
NPs: nanoparticles
VRP: verapamil
NVT: novantrone
NV@CaP-RGD: NVT- and VRP-loaded NPs
MDR: multidrug resistance
P-gp: P-glyco protein
MRP: multidrug resistance associated protein
BCRP: breast cancer resistance protein
