# Supplementary material for: Incorporation of drug efflux inhibitor and chemotherapeutic agent into an inorganic/organic platform for the effective treatment of multidrug resistant breast cancer
Source: J Nanobiotechnology. 2019 Dec 23;17:125. doi: 10.1186/s12951-019-0559-y (PMC6929438; doi:10.1186/s12951-019-0559-y)
Supplement: Supplementary file 1 — Additional file 1. Additional figures and experimental details. Figure S1. The average potential of NV@CaP-RGD was − 7.22 ± 0.39 mV. Figure S2. NV@CaP-RGD showed sustained-releasing and sequential-releasing pattern compared with that of unbounded NVT-VRP. Figure S3. The MCF7/MDR cells were incubated with VRP for various time (0.5 h, 1 h, 2 h, 3 h, 4 h) before adding NVT into it. Figure S4. The quantitative fluorescence intensity of Calcein AM at 6 h. Figure S5. The statistics of the internalization ability. Figure S6. The NV@CaP-RGD substantially promoted apoptosis and improved the therapeutic effect on MCF7 cells in vitro. Figure S7. The quantitative grayscale intensity of EKR and p-EKR proteins in MCF7 and MCF7/MDR cells. Figure S8. The quantitative grayscale intensity of efflux pumps proteins in MCF7 and MCF7/MDR cells. [file 12951_2019_559_MOESM1_ESM.docx]

**Incorporation of Drug Efflux Inhibitor and Chemotherapeutic Agent into an Inorganic/Organic Platform for the Effective treatment of Multidrug Resistant Breast Cancer**

Yang Dong^1^, Hongze Liao^2^, Jian Yu^1^, Hao Fu^1^, De Zhao^1^, Ke Gong^1^, Qi Wang^3,*^, and Yourong Duan^1,*^

^1^ State Key Laboratory of Oncogenes and Related Genes, Shanghai Cancer Institute, Renji Hospital, School of Medicine, Shanghai Jiao Tong University, Shanghai 200032, China.

^2^ Marine Drugs Research Center, Department of Pharmacy, State Key Laboratory of Oncogenes and Related Genes, Renji Hospital, School of Medicine, Shanghai Jiao Tong University, Shanghai 200127, China.

^3^ Key Laboratory for Advanced Materials and Institute of Fine Chemicals, Shanghai Key Laboratory of Functional Materials Chemistry, School of Chemistry and Molecular Engineering, East China University of Science and Technology, Shanghai 200237, China.

Corresponding authors:

Dr. Yourong Duan, State Key Laboratory of Oncogenes and Related Genes, Shanghai Cancer Institute, Renji Hospital, School of Medicine, Shanghai Jiao Tong University, Shanghai, China

E-mail: yrduan@shsci.org

Dr. Qi Wang, Key Laboratory for Advanced Materials and Institute of Fine Chemicals, Shanghai Key Laboratory of Functional Materials Chemistry, School of Chemistry and Molecular Engineering, East China University of Science and Technology, Shanghai, China

E-mail: wangqi@ecust.edu.cn

**Catalog**

**Experimental section**

**Additional Figures:**

**Fig S1.** The average potential of NV@CaP-RGD was -7.22 ± 0.39 mV.

**Fig S2.** NV@CaP-RGD showed sustained-releasing and sequential-releasing pattern compared with that of unbounded NVT-VRP.

**Fig S3.** The MCF7/MDR cells were incubated with VRP for various time (0.5 h, 1 h, 2 h, 3 h, 4 h) before adding NVT into it.

**Fig S4.** The quantitative fluorescence intensity of Calcein AM at 6 h.

**Fig S5.** The statistics of the internalization ability.

**Fig S6.** The NV@CaP-RGD substantially promoted apoptosis and improved the therapeutic effect on MCF7 cells *in vitro*.

**Fig S7.** The quantitative grayscale intensity of EKR and p-EKR proteins in MCF7 and MCF7/MDR cells.

**Fig S8.** The quantitative grayscale intensity of efflux pumps proteins in MCF7 and MCF7/MDR cells.

**Experimental section**

**Materials, cell lines and animals**

Phosphatidylserine (PS) was purchased from Shanghai Yiji Medicine & Chemical Co, Ltd. Poly-(ethylene glycol) methyl ether (PEG, Mn=2000), Boc-NH-PEG-COO-NHS and CaCl_2_ were obtained from Sigma-Aldrich, and RGD was purchased from Corner Stone Therapeutics (Shanghai), Ltd (Shanghai, China). EDC and CDI were purchased from Acros. (NH_4_)_2_HPO_4_ was obtained from Sinopharm Chemical Reagent Co., Ltd.

Novantrone (NVT) was purchased from Chongqing Carelife Pharmaceutical Co, Ltd. Verapamil (VRP) was purchased from Melonepharma Co, Ltd, Dalian. The annexin V-FITC (annexin V), ATP test kit and the propidium iodide (PI) detection kit were purchased from BD PharMingen, San Diego, CA. MTT and Calcein AM were obtained from Sigma-Aldrich. lysoTracker red probe and DAPI (4', 6-diamidino-2-phenylindole) were obtained from Beyotime Institute of Biotechnology.

MCF7 cells (human breast cancer cells) and MCF7/MDR cells (multidrug resistant cells) were obtained from the Type Culture Collection of the Chinese Academy of Sciences, Shanghai, China. FBS (fetal bovine serum) was procured from Gibco BRL. DMEM (dulbecco's modified eagle medium) was manufactured by HyClone. Nude BALB/c mice (6 weeks old, weighing 18 - 22 g) were provided from the Animal Experiment Centre of Shanghai Cancer Institute. All of the animal experimental procedures were performed according to the protocols approved by the Animal Care and Use Committee of the Shanghai Cancer Institute.

**Preparation of the codelivery nanoparticles (NV@CaP-RGD)**

NV@CaP-RGD was prepared by the bio-mineralization method. 20 mg PS-PEG-RGD and 10 mg NVT were dissolved in 5 mL ethanol, in which the drug film was formed by rotary evaporation, followed by the addition of CaCl_2_ solution (50 mL, 12 mM) to hydrate the film (pH 10). Then, an (NH_4_)_2_HPO_4_ solution (50 mL, 8 mM) was added dropwise into the solution. The white precipitate was collected and washed with centrifugation-redispersion cycles. Next, 10 mg VRP was dissolved in this dispersion system and mixed using an ultrasonic processor (JY92-II ultrasonic processor, Ningbo Scientz Biotechnology Co, Ltd. China). After stabilization and further sonication for another 2 min, the NV@CaP-RGD were collected by centrifugation at 3500 rpm for 30 min and freeze-dried.

To satisfy the demands of the different experiments, during the preparation of drug-loaded RGD-NPs, NVT was replaced by Rb (red fluorescent probe), FTIC (green fluorescent probe), Dir (1,1-dioctadecyl-3,3,3,3- tetramethylindotricarbocyaine iodide) or Cy5 (red fluorescent probe) to prepare Rb-VRP-RGD-NPs, FITC-VRP-RGD-NPs, Dir-VRP-RGD-NPs or Cy5-VRP-RGD-NPs, respectively. To ensure a greater reliability of these results, the VRP remained on the surface of the RGD-NPs.

**Characteristics of NV@CaP-RGD**

The size and surface potential of NPs was evaluated using the zetasizer IV analyzer (Malvern Zetasizer Nano ZS90, Malvern, UK). To observe the morphology of the nanoparticles, the NPs were observed by a transmission electron microscope (H-800; Hitachi, Japan). The DL% and EE% of VRP and NVT in NPs or RGD-NPs was determined after ultracentrifugation (30 min, 7500 g, 4 ℃). Unbounded VRP and NVT in the supernatant were diluted with the mobile phase and analyzed using HPLC assay. The Kromasil ODS-1 column (150 mm × 4.6 mm, 5 µm) was used to separate the samples. For NVT, the mobile phase was a mixture of methanol and 0.02 M monopotassium phosphate (36: 64, v/v; adjusted to pH 3.0 with phosphoric acid). The eluents were monitored at 599 nm (AUFS = 1). For VRP, the mobile phase was a mixture of methanol, triethylamine and sodium acetate buffer solution (55: 1: 45, V/V). The eluents were monitored at 278 nm (AUFS = 1). The injection volume was 20 µL and eluted at a flow rate of 1.0 mL min^-1^ at 30 ℃.

**Release profile of NV@CaP-RGD *in vitro***

Release profile of VRP and NVT from NPs and RGD-NPs *in vitro* was investigated using a dialysis method. Briefly, the appropriate amount of NV@CaP-RGD was suspended in the dialysis bag (Mw cutoff: 10000 - 12000; Millipore, USA) and immersed into the medium. 0.1 M of 1000 mL phosphate buffered saline solution (pH 7.4 and 5.5) were used as the medium, gently shaking at 37 ℃. Simultaneously, unbounded VRP-NVT was tested as the control. 2 mL sample was removed from the release medium and replaced with an equal volume of fresh phosphate buffered saline solution. The amount of VRP and NVT released in the supernatant was determined using HPLC.

**ATP-consuming assays and Calcein AM Assay for efflux pumps function**

MCF7 or MCF7/MDR cells were seeded in 6-well plates at a density of 2 × 10^5^ cells well^-1^ and incubated overnight. Various doses of NPs and NPs-RGD in serum-free DMEM were added to cells and incubated at 37 ℃ for 4 h. Following treatment, the cells were washed twice with serum-free DMEM and measured using an ATP Assay Kit and normalized using protein content.

MCF7 or MCF7/MDR cells were seeded in black 96-well plates at a density of 1 × 10^5^ cells well^-1^ overnight and treated with 50 µL of VRP-NVT (1 µg mL^-1^), NV@CaP (1 µg mL^-1^) or NV@CaP-RGD (1 µg mL^-1^). After 4 h at 37 ℃, 50 µL of 0.25 µM Calcein AM were added into each well and the fluorescence was immediately measured after 1 h using a microplate reader at 485/589 excitation/emission at room temperature. The % relative fluorescence in the cells was expressed as:

% Relative Fluorescence (FL) = [(FLtreatment – FLnontreatment)/ FLnontreatment] × 100%

To evaluate the cell images, the Calcien fluorescence was immediately measured using fluorescence microscopy.

**Cellular uptake assay**

The cellular uptake of NPs was assayed using invert fluorescence microscopy for direct images and HPLC for accurate internalization. Three freeze/thaw cycles of the suspensions were performed to release the intracellular drug, and acetonitrile and methanol (volume ratio = 10 : 90, 0.5 mol L^-1^ HCl) was added to extract the drugs. The HPLC system was used to analyze the drug concentrations. The cellular uptake of Rb was assayed using invert fluorescence microscopy.

**Lysosome escape assay**

MCF7 or MCF7/MDR cells were cultured in confocal microscopy (NEST, China) and incubated with unbound VRP-FITC, FITC-VRP@CaP or FITC-VRP@CaP-RGD for 24 h. The lysoTracker red probe (75 nM) was used to label the lysosome for 30 min. Subsequently, the nucleus was labeled with DAPI, and an FV-1200 Olympus confocal microscope was utilized to observe the intracellular distribution of FITC.

**Western blotting**

BCA protein assay (Thermo Fisher Scientific, Australia) was used to determine protein concentration. Proteins were separated by SDS-polyacrylamide gel electrophoresis (SDS–PAGE) and transferred onto a polyvinylidene difluoride (PVDF) membrane (Bio-Rad, USA) under constant 270 mA for 70 min and then the film was incubated with P-gp, MRP, BCRP, ERK and p-ERK antibodies. Immunoblots were detected by horseradish peroxidase (HRP) conjugated anti-mice/rabbit IgG using a chem-iluminescence kit (Agilent, USA) and visualized by a bio-rad imaging system (Bio-Rad, USA). The nitrocellulose film was incubated with GAPDH antibodies as control. Data are presented as the relative density of protein bands normalized to GAPDH.

**Detection of treatment effects *in vitro***

The MCF7/MDR cells were treated with unbound VRP and NVT (1 µg mL^-1^), NV@CaP (1 µg mL^-1^) and NV@CaP-RGD (1 µg mL^-1^) for 72 h. Annexin V-PI with flow cytometry analysis using FACScan (Becton Dickinson) was used to assess cell apoptosis.

MTT assays were performed to detect cell toxicity. The MCF7 or MCF7/MDR cells were seeded in 96-well culture plates at a density of 1 × 10^4^ cells well^-1^. In addition, the MTT assays were performed at 72 h with a series of concentrations of samples.

JC-1 was used to determine the mitochondrial membrane potential. MCF7 or MCF7/MDR cells were exposed to unbound VRP-NVT (1 µg mL^-1^), NV@CaP (1 µg mL^-1^) and NV@CaP-RGD (1 µg mL^-1^) for 24 h. Then, JC-1 (5 µg mL^-1^) was added, and the cells were imaged on a Ti-E microscope (Nikon Co., Japan) with an UltraVIEW Vox (PerkinElmer Inc., USA) confocal attachment using a 60 × 1.4 NA plan apochromat oil immersion lens or digested and subjected to flow cytometry analysis using FACScan (Becton Dickinson). The JC-1 monomer was detected in the FITC emission channel (522 - 535 nm), and the JC-1 aggregate was measured in the RFP (red fluorescence) emission channel (560 - 615 nm) with the same laser excitation of 488 nm. Note: Control +: carbonylcyanide-m- chlorophenylhydrazone, Control −: nontreated group.

**Live imaging using *in vivo* fluorescence imaging system**

The mice were divided into three groups randomly (n = 3). Unbound Dir, Dir-NPs and Dir-NPs-RGD were administered by intravenous injection when the volume of the tumor reached 100 mm^3^. Then, at 2, 4, 8, 24 and 48 h post-injection, the nude mice were anesthetized, and the fluorescence was observed using an *in vivo* imaging apparatus (LB983, Berthold Technologies Gmbh & Co. KG, Bad Wildbad, Germany). After the experiment, the nude mice were separately sacrificed. The heart, liver, spleen, lung, kidney, brain and tumor were harvested and observed (Ex was 649 nm, Em was 666 nm). Note: the heart, liver, spleen, lung and kidney were observed together, and the tumor tissue was observed separately.

**Tumor growth inhibition study *in vivo***

When the tumor volume was approximately 100 mm^3^, the mice were randomly divided into 4 groups and treated with different therapies: PBS solution, unbound NVT and VRP (both VRP and NVT are 2 mg kg^-1^), NV@CaP (2 mg kg^-1^), and NV@CaP-RGD (2 mg kg^-1^) through intravenous injection every 4 days for 4 weeks. The body weight and tumor size of each mouse were measured every other day. The diameter of the tumors was measured using a Vernier caliper. The estimated tumor volume was calculated using the formula: volume= 0.5 × length × width^2^. After the final injection, all tumors were collected for further analysis using H&E staining and TUNEL (terminal deoxynucleotidyl transferase dUTP nick end labeling) staining.

**Safety evaluation**

After the final injection, serum samples were collected by retro-orbital bleeding and measured to assess hepatic and renal damage. After the mice were sacrificed, the major organs, such as heart, liver, spleen, lung and kidney of each mouse were collected, fixed with formalin and processed for H&E staining to evaluate toxicity.

**Statistical analysis**

All statistical analyses were performed using SPSS version 21.0 (SPSS Inc., Chicago, IL, USA). Data are reported as the mean ± SD. The experimental data were statistically analyzed by using the t-test between independent samples. P values were considered statistically significant when less than 0.05.

**Additional Figures:**


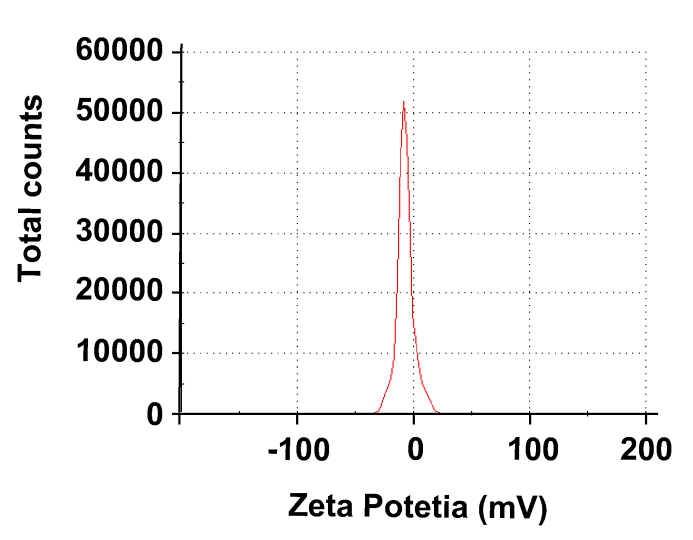


**Fig S1** The average potential of NV@CaP-RGD was -7.22 ± 0.39 mV.


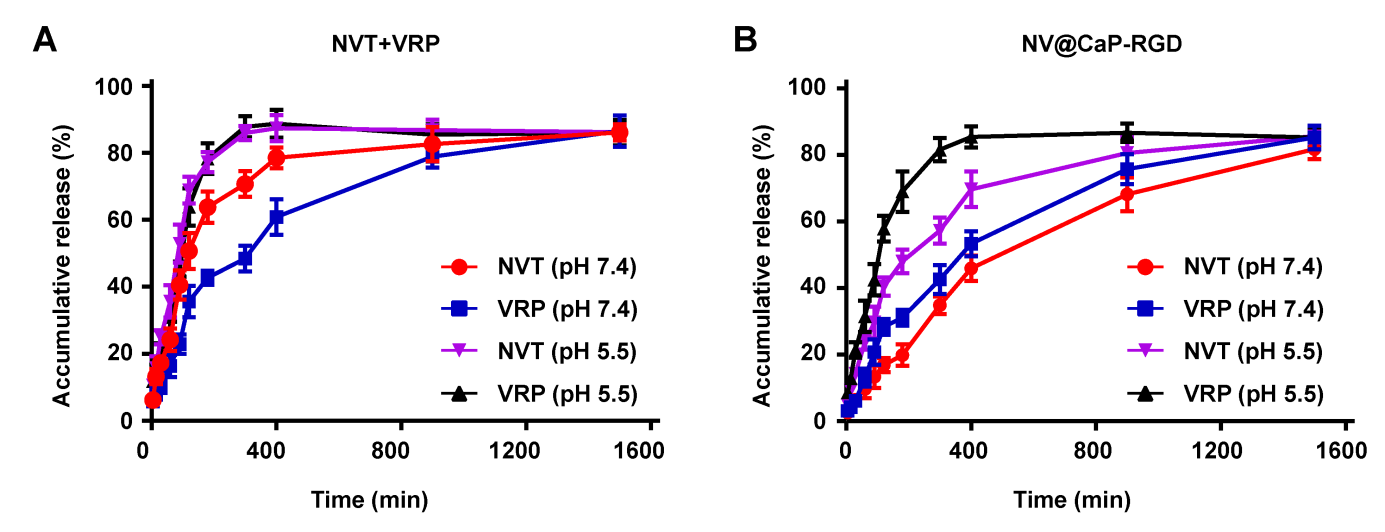


**Fig S2** NV@CaP-RGD showed sustained-releasing and sequential-releasing pattern compared with that of unbounded NVT-VRP. A) The drug release profiles of unbound VRP-NVT at pH 5.5 and 7.4. The accumulated amounts of NVT and VRP released from unbound drug solutions respectively reached a maximum at 400 min and 1000 min at pH 7.4, comparing to 400 min at pH 5.5. B) The drug release profile of NV@CaP-RGD at pH 5.5 and 7.4. NVT and VRP were released from the NPs sequentially at pH 5.5.


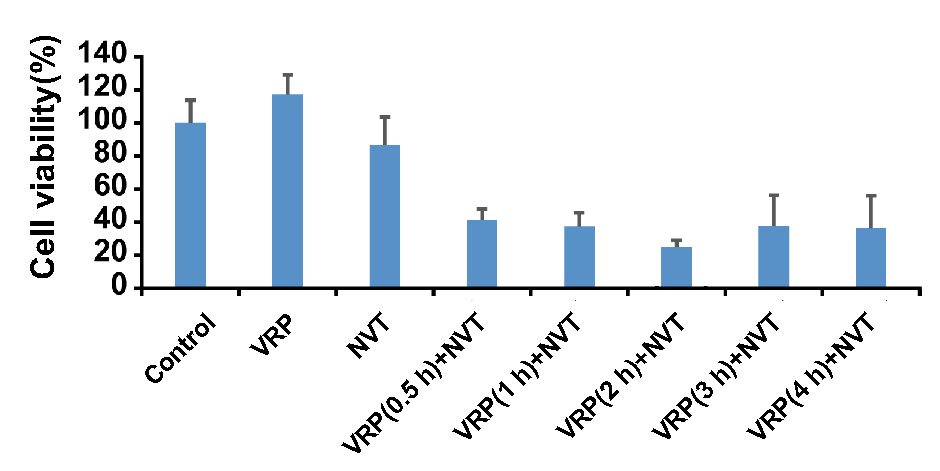


**Fig S3** The MCF7/MDR cells were incubated with VRP for various time periods (0.5 h, 1 h, 2 h, 3 h, 4 h) before adding NVT into it. After pretreated with VRP (1 μg mL^-1^) for various time periods (0.5 h, 1 h, 2 h, 3 h, 4 h), MCF7/MDR cells were incubated with NVT (1 μg mL^-1^) for 72 h. The greater lower cell viability was obtained after treated with VRP with 0.5 h, 1 h, 2 h, 3 h, 4 h, indicating the most suitable interval between VRP and NVT was longer than 0.5 h.


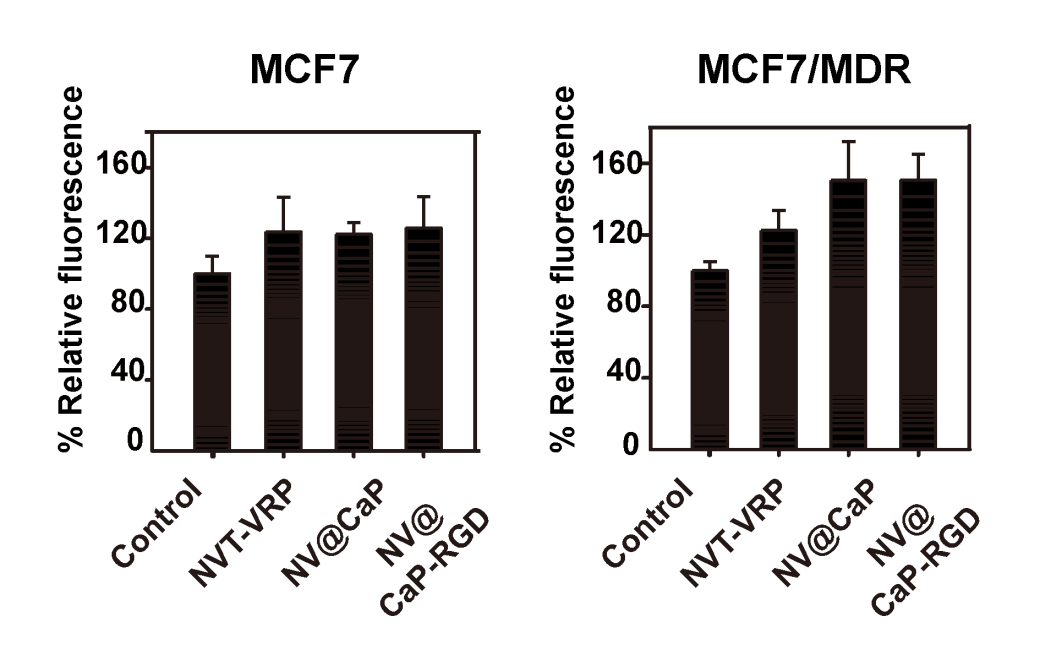


**Fig S4** The quantitative fluorescence intensity of Calcein AM at 6 h.


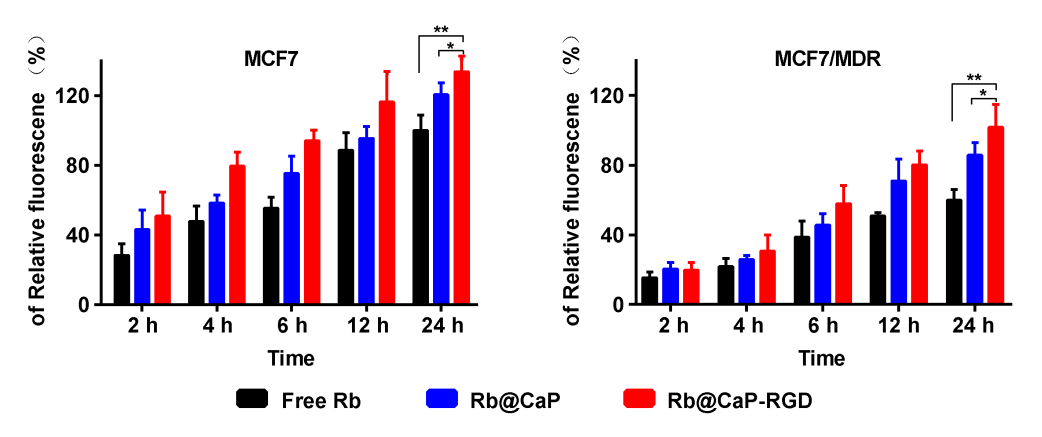


**Fig S5** The statistics of the internalization ability of Free Rb, Rb@CaP and Rb@CaP-RGD by breast cancer cells. Note: **P < 0.01; *P < 0.05.


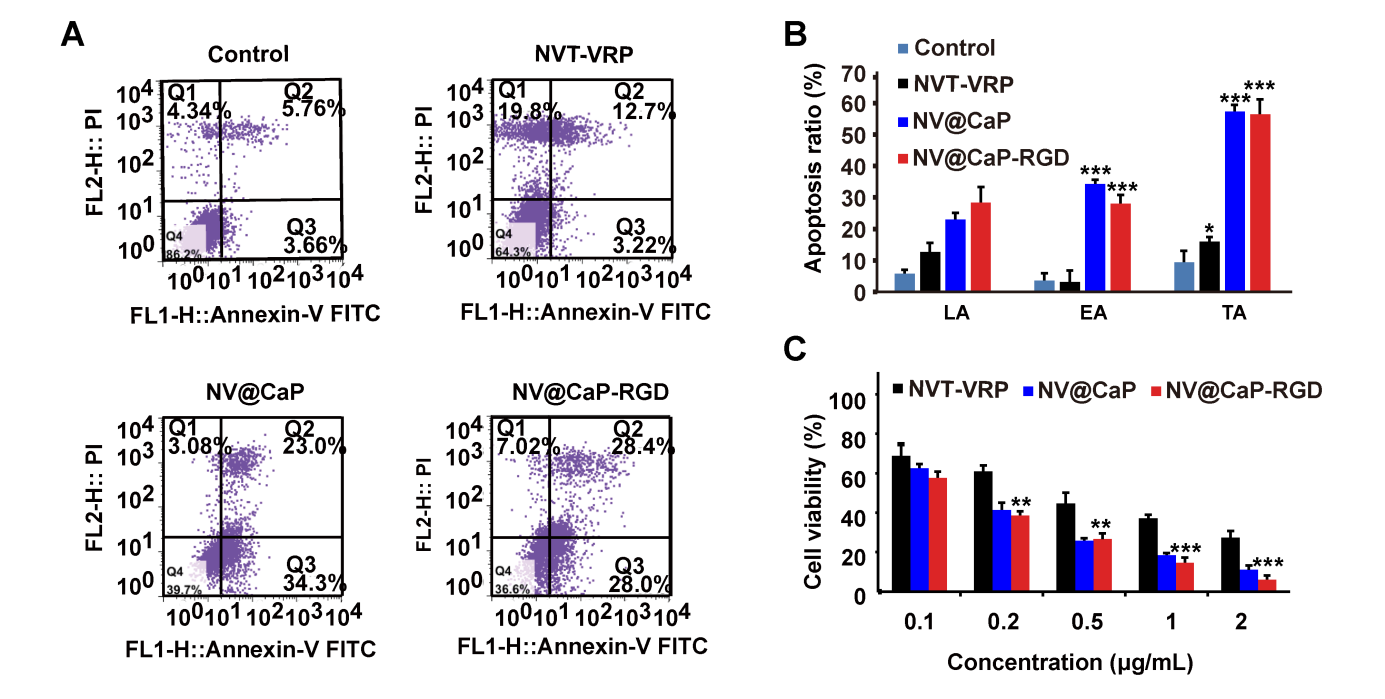


**Fig S6** The NV@CaP-RGD substantially promoted apoptosis and improved the therapeutic effect on MCF7 cells *in vitro*. A) Annexin V/PI double staining assay was used to evaluate the apoptotic efficiency of MCF7 tumor cells treated with unbounded VRP-NVT, NV@CaP-RGD and NV@CaP for 72 h. B) The quantitative analysis of apoptosis ratio. The co-delivery of VRP and NVT by RGD-NPs had the best synergistic effect on inducing apoptosis of MCF7 cells. Note: EA means early apoptosis, LA means late apoptosis, TA means total apoptosis. C) Cell viability of MCF7 cells with the treatment of various concentrations of unbounded VRP and NVT (black column), NV@CaP (blue column) and NV@CaP-RGD (red column) for 72 h (*P < 0.05, **P < 0.01, ***P < 0.001 compared with control group). The cell viability of MCF7 cells significantly decreased in NV@CaP-RGD and NV@CaP groups, suggesting NV@CaP-RGD was able to effectively inhibit the viability of tumor cells at the cellular level.


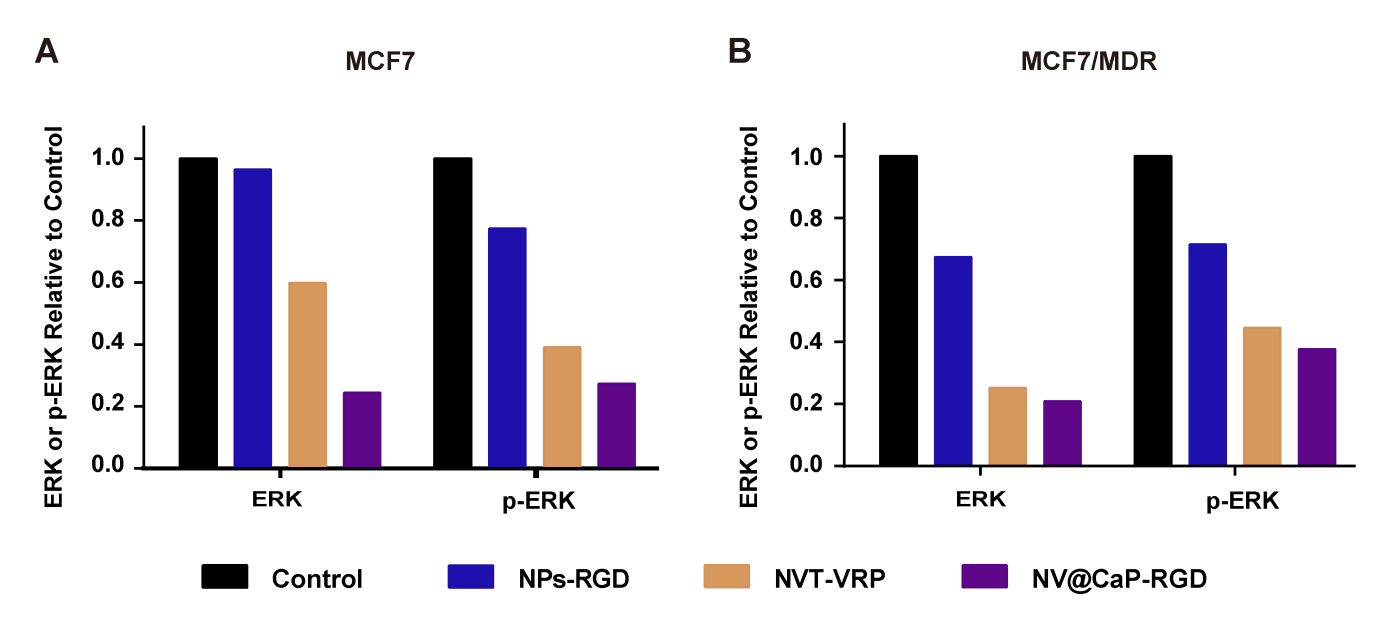


**Fig S7** The quantitative grayscale intensity of EKR and p-EKR proteins in A) MCF7 and B) MCF7/MDR cells. Downregulation of EKR and p-EKR proteins in MCF7 and MCF7/MDR cells indicated that NV@CaP-RGD NPs were able to effectively inhibit breast cancer cells proliferation *via* interference of the ERK signaling transduction pathway.


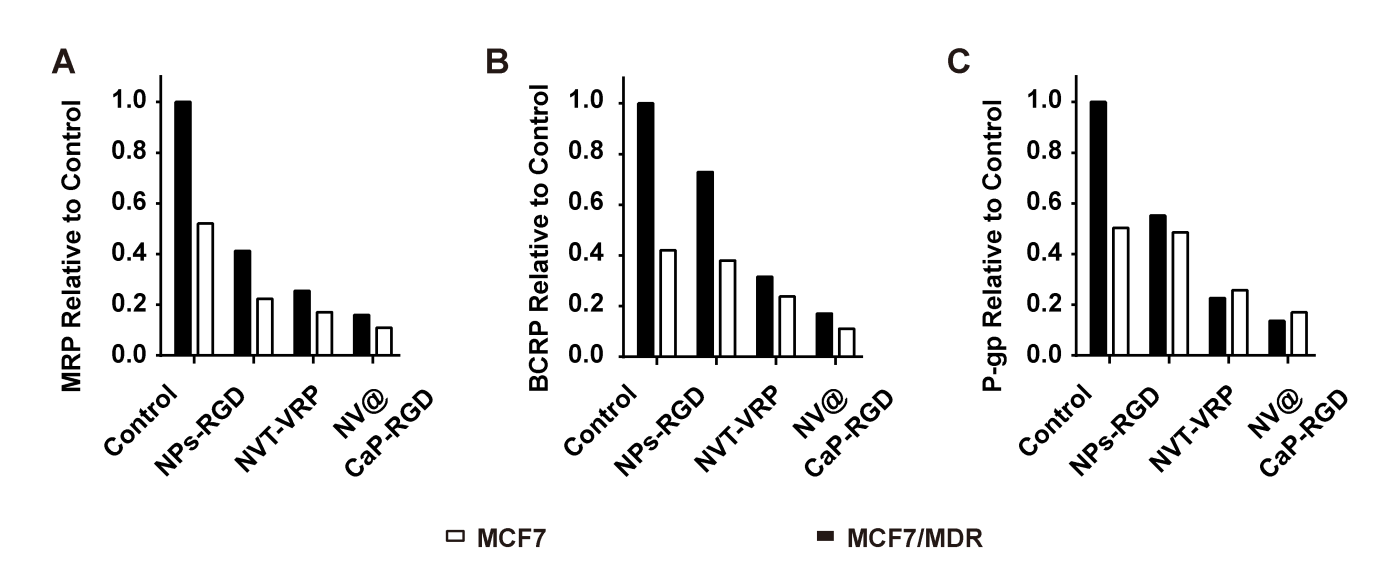


**Fig S8** The quantitative grayscale intensity of efflux pumps proteins in MCF7 and MCF7/MDR cells. Western blotting analysis showed that NV@CaP-RGD significantly decreased the expression of A) MRP, B) BCRP and C) P-gp proteins.
